# Nucleocapsid Promotes Localization of HIV-1 Gag to Uropods That Participate in Virological Synapses between T Cells

**DOI:** 10.1371/journal.ppat.1001167

**Published:** 2010-10-28

**Authors:** G. Nicholas Llewellyn, Ian B. Hogue, Jonathan R. Grover, Akira Ono

**Affiliations:** 1 Cellular and Molecular Biology Program, University of Michigan Medical School, Ann Arbor, Michigan, United States of America; 2 Department of Microbiology and Immunology, University of Michigan Medical School, Ann Arbor, Michigan, United States of America; Northwestern University, United States of America

## Abstract

T cells adopt a polarized morphology in lymphoid organs, where cell-to-cell transmission of HIV-1 is likely frequent. However, despite the importance of understanding virus spread in vivo, little is known about the HIV-1 life cycle, particularly its late phase, in polarized T cells. Polarized T cells form two ends, the leading edge at the front and a protrusion called a uropod at the rear. Using multiple uropod markers, we observed that HIV-1 Gag localizes to the uropod in polarized T cells. Infected T cells formed contacts with uninfected target T cells preferentially via HIV-1 Gag-containing uropods compared to leading edges that lack plasma-membrane-associated Gag. Cell contacts enriched in Gag and CD4, which define the virological synapse (VS), are also enriched in uropod markers. These results indicate that Gag-laden uropods participate in the formation and/or structure of the VS, which likely plays a key role in cell-to-cell transmission of HIV-1. Consistent with this notion, a myosin light chain kinase inhibitor, which disrupts uropods, reduced virus particle transfer from infected T cells to target T cells. Mechanistically, we observed that Gag copatches with antibody-crosslinked uropod markers even in non-polarized cells, suggesting an association of Gag with uropod-specific microdomains that carry Gag to uropods. Finally, we determined that localization of Gag to the uropod depends on higher-order clustering driven by its NC domain. Taken together, these results support a model in which NC-dependent Gag accumulation to uropods establishes a preformed platform that later constitutes T-cell-T-cell contacts at which HIV-1 virus transfer occurs.

## Author Summary

CD4^+^ T cells are natural targets of HIV-1. Efficient spread of HIV-1 from infected T cells to uninfected T cells is thought to occur via cell-cell contact structures. One of these structures is a virological synapse where both viral and cellular proteins have been shown to localize specifically. However, the steps leading to the formation of a virological synapse remain unknown. It has been observed that T cells adopt a polarized morphology in lymph nodes where cell-to-cell virus transmission is likely to occur frequently. In this study, we show that in polarized T cells, the primary viral structural protein Gag accumulates to the plasma membrane of a rear end structure called a uropod. We found that Gag multimerization, driven by its nucleocapsid domain, is essential for Gag localization to uropods and that HIV-1-laden uropods mediate contact with target cells and can become part of the virological synapse. Our findings elucidated a series of molecular events leading to formation of HIV-1-transferring cell contacts and support a model in which the uropod acts as a preformed platform that constitutes a virological synapse after cell-cell contact.

## Introduction

One of the primary natural targets of HIV-1 is the T cell. HIV-1 spread between infected and uninfected T cells likely occurs frequently in densely packed environments such as lymph nodes *in vivo*. Two-photon imaging studies have shown that a majority of T cells in lymph nodes are highly motile and have a polarized morphology [1], [2], [3], [4], [5], [6]. Therefore, it is likely that, in lymphoid organs, HIV-1 replicates within and is transmitted by polarized T cells. However, the life cycle of HIV-1 in polarized T cells has not been examined in detail.

HIV-1 assembly occurs at the plasma membrane and is driven by the HIV-1 polyprotein Gag. Gag is the primary structural protein of retroviruses, including HIV-1, and is both necessary and sufficient for formation of virus-like particles [7]. HIV-1 Gag is composed of four structural domains: matrix (MA), capsid (CA), nucleocapsid (NC) and p6. MA mediates Gag targeting and binding to the plasma membrane, primarily through the myristoyl group on the N terminus of MA, which inserts into the plasma membrane, as well as MA basic amino acids that interact with phosphatidylinositol-4,5-bisphosphate [PI(4,5)P_2_], a plasma-membrane-specific phospholipid [8], [9], [10], [11], [12], [13], [14], [15], [16], [17], [18], [19], [20]. CA mediates Gag dimerization through an interface in its C terminal domain (CTD), in which amino acids W184 and M185 play key roles [21], [22], [23], [24], [25], [26], [27], [28], [29], [30], [31], [32], [33]. NC binds specifically to the viral genomic RNA, which is essential for packaging viral genomes into virions [34]. In addition, NC contributes to multimerization of Gag, whereby RNA is thought to serve as a scaffold [21], [25], [28], [35], [36], [37], [38], [39], [40], [41], [42], [43]. p6 contains peptide sequences that recruit cellular endosomal sorting complex required for transport (ESCRT) proteins, which facilitate the release of virus particles [44], [45], [46].

A polarized T cell forms a leading edge at the front and a protrusion called a uropod at the rear [47], [48], [49]. There are several proteins known to be enriched in the uropod, including intercellular adhesion molecule (ICAM)-1, -2, and -3, P-selectin glycoprotein ligand (PSGL)-1, CD43, and CD44 [50], [51]. The microtubule organizing center (MTOC) is also known to localize to the base of the uropod [49], [52]. Previous studies have observed that in T cells and monocytes, HIV-1 proteins localize to a cell protrusion, which resembles a uropod [53], [54], [55], [56], [57]. Furthermore, virus particles are enriched in several uropod-associated proteins, such as ICAM-1, ICAM-2, CD43 and CD44 [58], [59]. A raft-associated lipid known to localize to uropods, GM1 [60], also associates with virus particles [54], [61], [62]. Altogether, these observations suggest that uropods potentially serve as sites of virus assembly in polarized T cells. However, the nature of Gag localization in polarized T cells and its significance to virus spread have yet to be fully determined.

T cell uropods have been shown to mediate contact between T cells and other cells, which is consistent with the observation of adhesion molecule enrichment in uropods [63], [64], [65]. Thus, it is possible that HIV-1 accumulation at the uropod may play a role in cell-to-cell transmission. Cell-to-cell transmission is ten to several thousand times more efficient than cell-free transmission [53], [57], [66], [67], [68], [69], [70], [71]. Recent studies have described specific cell contact structures that facilitate cell-to-cell transmission [67], [72], [73], [74], [75], [76], [77], [78], [79], [80], [81], [82], [83], [84], [85], [86]. Live cell imaging studies have revealed that particles of HIV-1 and murine leukemia virus (MLV) are transferred from infected cells to uninfected target cells along the surface of filamentous extensions called membrane nanotubes and cytonemes [80], [81], [82], [87], [88]. Virological synapses (VS), which appear to structurally resemble immunological synapses [77], [78], [89], [90], [91], [92], [93], [94], [95], [96], [97], are also thought to facilitate the direct transfer of budding virus particles from one cell to another [53], [67], [71], [78], [80], [95]. However, the mechanisms leading to the establishment of these transmission routes, especially the VS, remain to be elucidated.

In this study, we determined unambiguously that the uropod is the cell structure to which membrane-associated Gag accumulates in polarized T cells. Gag-containing uropods mediated frequent contact with uninfected target cells. Virtually all observed VS, defined by accumulation of CD4 and Gag to cell contacts, showed enrichment of the uropod marker CD43, suggesting a major role for HIV-1 localization to the uropod in virus spread. Consistent with this possibility, upon disruption of uropod formation, cell-to-cell transfer of HIV-1 was significantly reduced. Gag on the cell surface copatched strongly with uropod markers not only in polarized T cells but also in non-polarized T cells. Gag-containing patches dispersed on the membrane of non-polarized cells appeared to laterally move and concentrate at the uropod when cells became polarized. These patches maintained colocalization with uropod markers, suggesting that uropod-directed microdomains play a role in polarized Gag localization. Uropod localization of Gag required higher-order multimerization or clustering mediated by NC. These findings strongly support that multimerization-dependent Gag localization to uropods represents one mechanism by which the VS is formed.

## Results

### Gag localizes to uropods in polarized T cells

**Figure 1:**
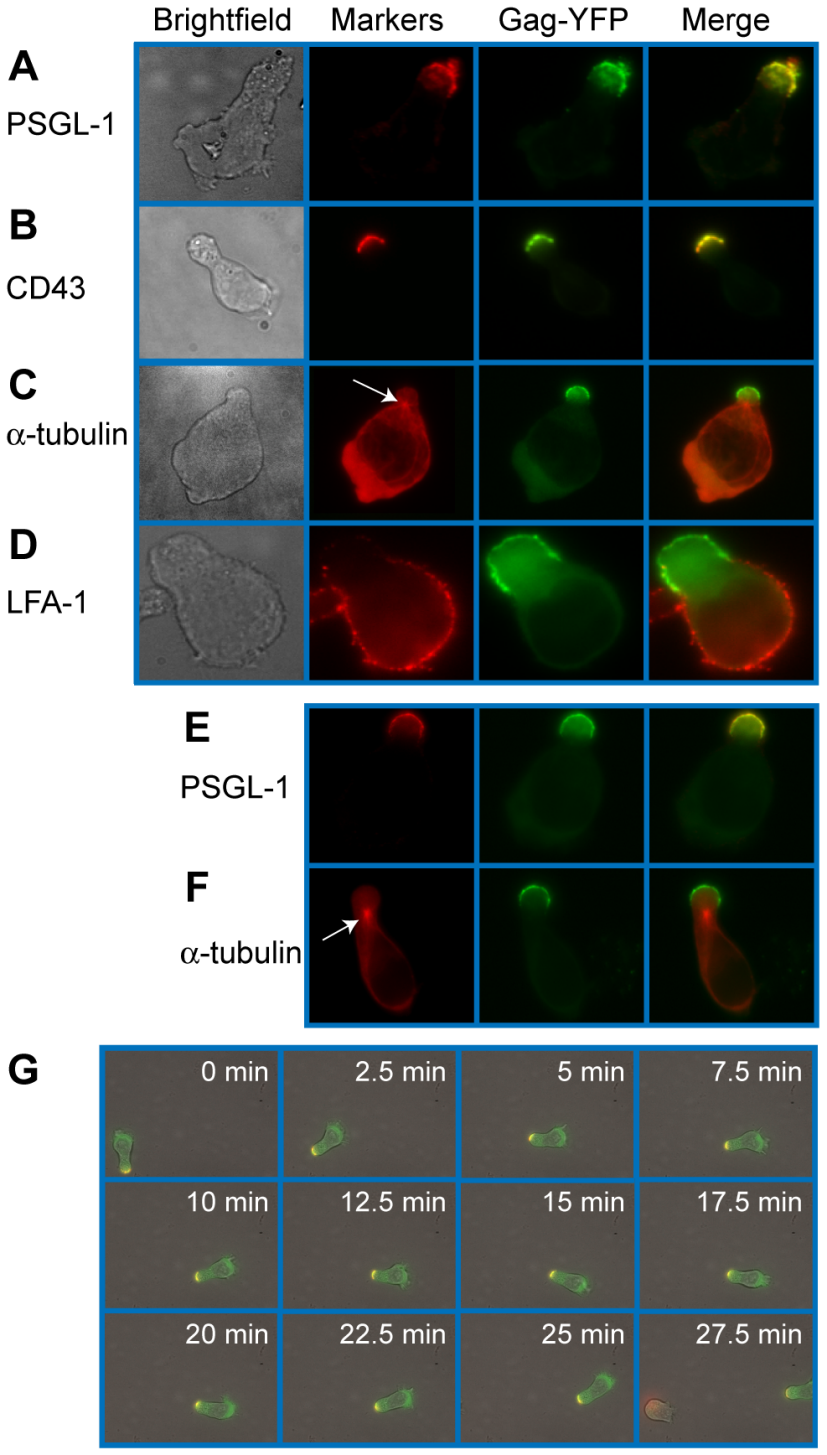
Gag stably localizes to the uropod in polarized T cells. Primary T cells (**A–D**) and P2 cells (**E–F**) expressing Gag-YFP (green) were immunostained for uropod and non-uropod markers as described in the Materials and Methods section, and observed using an epifluorescence microscope. Uropods were identified by the presence of PSGL-1 (**A** and **E**) and CD43 (**B**) as well as by the location of the MTOC determined by immunostaining with anti- α-tubulin (**C** and **F**, arrows). LFA-1 (**D**) is a known non-uropod marker and served as a negative control. **G**) Cells expressing Gag-YFP (green) were immunostained with anti-PSGL-1 prelabeled by AlexaFluor-594-conjugated anti-mouse IgG (red). Images were acquired every 30 s for 30 min as the polarized cell migrated through the field. Yellow color indicates colocalization of PSGL-1 and Gag-YFP.

To examine Gag localization in polarized T cells, we expressed a YFP-tagged Gag (Gag-YFP) in either primary T cells or in a polarized T cell line, P2. To express Gag-YFP, T cells were infected with VSV-G-pseudotyped HIV-1 that encodes Gag-YFP. Two days post-infection, cells were immunostained for uropod markers PSGL-1 or CD43 (Figure 1A and B). Alternatively, the MTOC, which localizes to the base of the uropod, was detected using anti-α-tubulin (Figure 1C). In both primary CD4^+^ T cells and P2 cells, approximately 50-60% of cells showed polarized morphology, and infection with VSV-G-pseudotyped HIV-1 did not substantially alter the percentage of polarized cells (Table 1). Primary CD4^+^ T cells expressing Gag-YFP showed strong colocalization of Gag on the plasma membrane with both uropod markers PSGL-1 and CD43, as well as co-polarization with the MTOC in virtually all Gag-positive cells with uropods (Figure 1A–C and Table 2). In contrast, Gag showed segregation from LFA-1, a non-uropod-associated protein [98] (Figure 1D). Similar to primary T cells, P2 cells also showed strong colocalization of PSGL-1 and Gag-YFP, as well as co-polarization of the MTOC and Gag-YFP (Figure 1E and F and Table 2). In these cells, plasma-membrane-associated Gag was highly polarized and detected only in the uropod region (Gag polarization was quantitatively analyzed as shown below). Similarly, untagged Gag detected at the plasma membrane using anti-Gag antibodies also showed strong colocalization with uropod markers (Figure S1). These results indicate that Gag localizes to uropods in polarized T cells. To determine whether uropod localization of Gag-YFP is stable, we performed live cell analysis of primary T cells expressing Gag-YFP. We observed that Gag-YFP maintains localization in the uropod during T cell migration for a minimum of almost 30 min (Figure 1G and Movie S1).

**Table 1:** Quantification of T Cell Polarization.

	Gag-Expressing Cells			Non-Gag-Expressing Cells		
	Polarized (% of total)	Unpolarized (% of total)	Total	Polarized (% of total)	Unpolarized (% of total)	Total
Primary CD4^+^ T cells	253 (64.4%)	140 (35.6%)	393	530 (56.6%)	406 (43.4%)	936
P2 cells	176 (51.9%)	163 (48.1%)	339	97 (47.1%)	109 (52.9%)	206

Primary CD4^+^ T cells and P2 cells infected with VSV-G-pseudotyped HIV-1 encoding Gag-YFP were cultured for two days, fixed, immunostained for cellular proteins, and examined by fluorescence microscopy. Gag-YFP-positive and -negative cells were categorized based on cell morphology, and cells in each category were counted. Cells with circularity below 0.8 (see Materials and Methods) were categorized as polarized cells.

**Table 2:** Copolarization of Gag with Cellular Markers.

	Marker	Gag Polarized		Gag Not Polarized	Total Cell Number
		Copolarized with Marker	Not Copolarized with Marker		
Primary CD4^+^ T cells	PSGL-1	96%	2%	2%	58
	CD43	100%	0%	0%	58
	MTOC	96%	1%	3%	69
	LFA-1	13%	86%	1%	69
P2 cells	PSGL-1	79%	19%^a^	2%	63
	MTOC	83%	9%^b^	8%	111

Primary CD4^+^ T cells and P2 cells infected with VSV-G-pseudotyped HIV-1 encoding Gag-YFP were cultured for two days, immunostained for cellular proteins, and examined by fluorescence microscopy (see Materials and Methods). Gag-YFP-positive cells that were categorized as polarized cells based on cell morphology were further examined for polarized localization of Gag-YFP and copolarization of Gag-YFP with cellular markers.

a In P2 cells where Gag did not copolarize with PSGL-1, PSGL-1 did not show polarized localization.

b In P2 cells where Gag did not copolarize with MTOC, MTOC localized near the center of cells.

**Figure 2:**
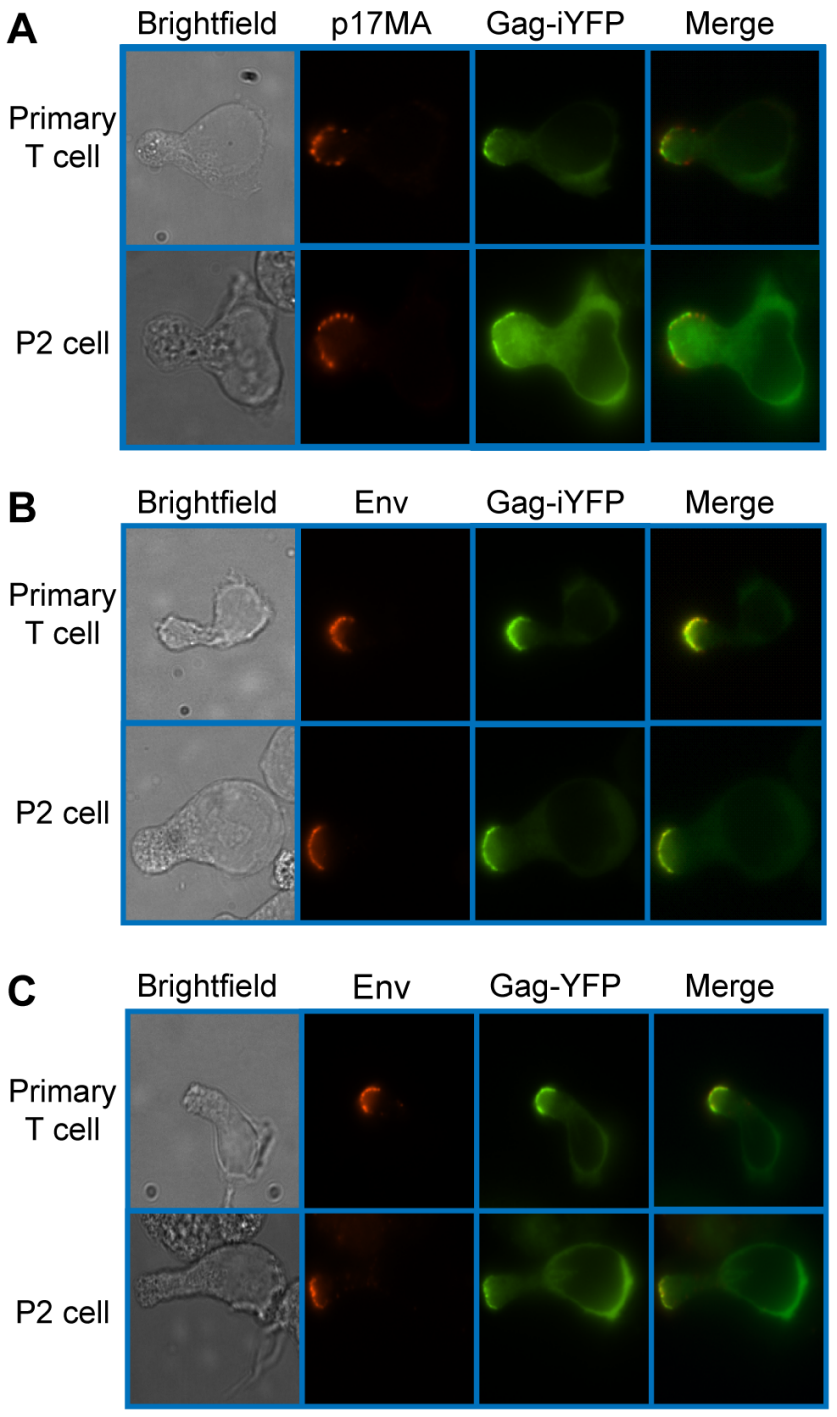
Mature Gag and Env localize to the uropod. Primary CD4^+^ T cells and P2 cells were infected with a VSV-G-pseudotyped HIV-1 encoding Gag-iYFP (green) (**A** and **B**) or Gag-YFP (green) (**C**). **A**) For detection of mature Gag, cells were fixed, permeabilized, and immunostained with anti-p17MA (red) as described in Materials and Methods and observed with an epifluorescence microscope. **B**) and **C**) For detection of Env on the cell surface, infected cells were incubated with anti-gp120 (IgG1 b12) and subsequently with AlexaFluor-594-conjugated anti-human IgG prior to fixation as described in Materials and Methods.

To determine whether uropod-associated Gag is able to form mature particles, P2 and primary CD4^+^ T cells were infected with VSV-G-pseudotyped HIV-1 encoding Gag-iYFP. This Gag derivative contains YFP inserted between MA and CA and forms mature Gag proteins and free YFP upon cleavage by viral protease [99]. When cells expressing Gag-iYFP were immunostained with an anti-p17MA antibody, which only recognizes the mature, cleaved matrix domain of Gag [100], [101], the YFP signal was observed to colocalize substantially with p17MA signal at the uropod (Figure 2A and Table 3). We also observed that both Gag-iYFP and Gag-YFP colocalize well with HIV-1 Env in the uropod (Figure 2B and Table 3). These results suggest that at least a subset of Gag localized at uropods is capable of forming Env-containing virus particles that undergo Gag processing essential for virion maturation. It should be noted that, similar to previous studies [61], [96], we performed immunostaining of Env prior to fixation. Thus, the possibility of antibody crosslinking playing a role in Env localization should be considered.

**Table 3:** Colocalization of YFP-Tagged Gag with Antibody-Detected Viral Proteins.

YFP-Tagged Gag	Antibody	Cell Type	YFP colocalized with antibody signal	YFP not colocalized with antibody signal	Total Cell Number
Gag-iYFP	Anti-p17MA	Primary CD4^+^ T cells	92%	8%	51
		P2 cells	88%	12%	52
	Anti-gp120 (b12)	Primary CD4^+^ T cells	92%	8%	50
		P2 cells	92%	8%	61
Gag-YFP	Anti-gp120 (b12)	Primary CD4^+^ T cells	91%	9%	56
		P2 cells	93%	7%	75

Primary CD4^+^ T cells and P2 cells infected with VSV-G-pseudotyped HIV-1 encoding Gag-iYFP or Gag-YFP were cultured for two days, immunostained for viral proteins, and examined by fluorescence microscopy. YFP-positive polarized cells were examined for colocalization of the cell surface YFP-tagged Gag proteins and viral antigens detected by anti-p17MA or anti-gp120 (b12).

### Uropods mediate contact between infected and target T cells

**Figure 3:**
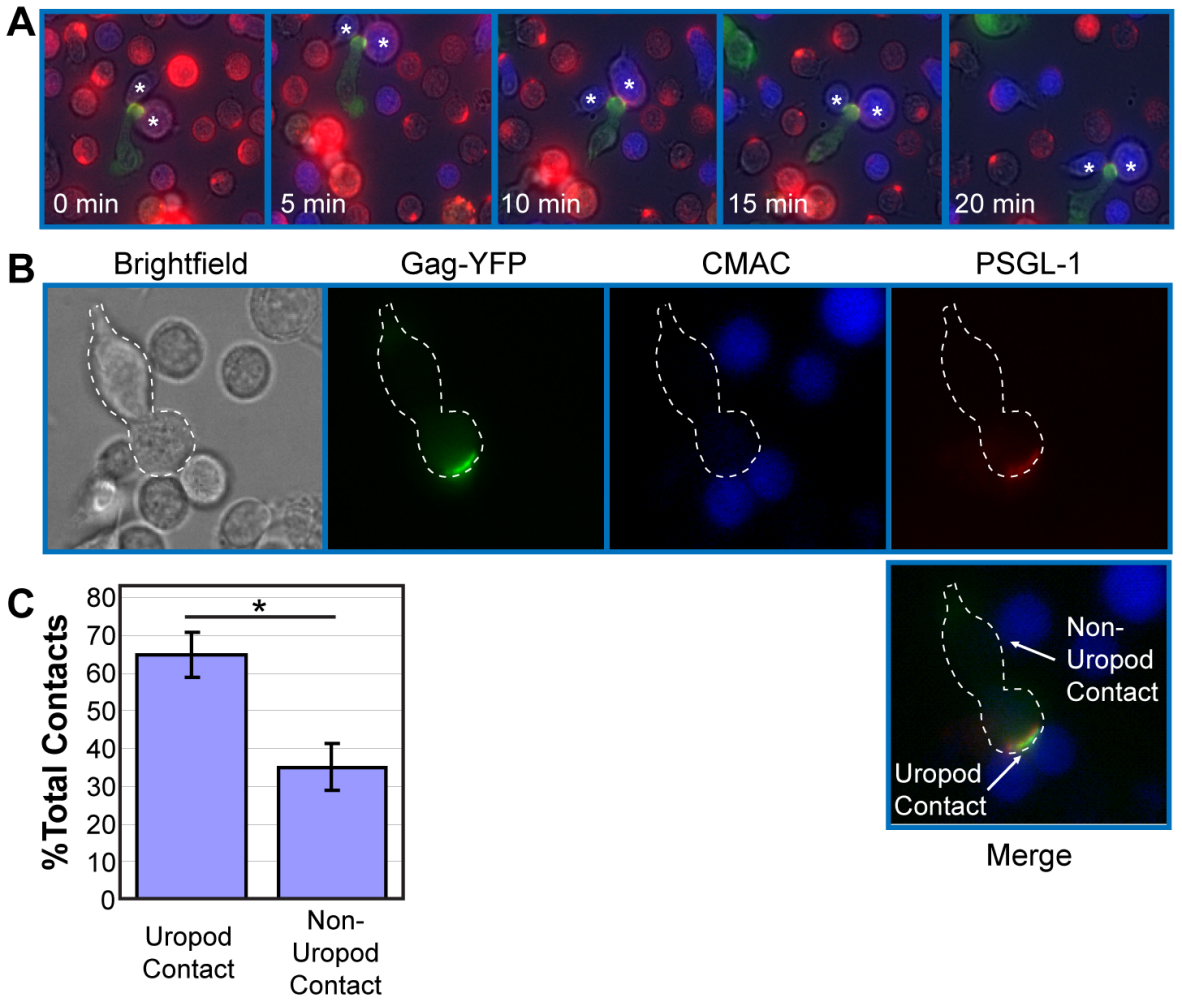
Infected polarized T cells form contacts with target cells via their uropods. **A**) Primary T cells expressing Gag-YFP (green) were immunostained with an anti-PSGL-1 antibody (red) as described in Figure 1G and cocultured with fresh primary T cells from the same donor stained with the fluorescent dye CMAC (blue). Regions of colocalization between Gag and PSGL-1 are shown in yellow. Live cell images were taken every 30 s for 20 min. A series of images with 5-min intervals is shown. Note that the uropod, enriched in Gag-YFP and PSGL-1, mediates stable contacts with target cells (*). **B**) Examples of uropod- and non-uropod-mediated contacts between a Gag-YFP expressing primary T cell (dotted white outline) with CMAC-labeled primary T cells are shown. **C**) Uropod-mediated and non-uropod-mediated contacts were counted for a total of 74 polarized Gag-YFP-positive cells contacting CMAC-labeled cells in two independent experiments. P values were determined using Student's t test. *, P<0.05.

Uropods in uninfected T cells have been shown to mediate contact between T cells and other cells [63], [65]. Therefore, accumulation of Gag to, and particle formation at, the uropod may facilitate cell-to-cell transmission of HIV-1. To examine whether contact of HIV-1-infected T cells with target T cells is preferentially mediated by uropods, we performed live cell imaging experiments. Fresh target primary T cells were stained with a blue fluorescent dye, CMAC, and cocultured with Gag-YFP-expressing primary T cells. This coculture was then immunostained with anti-PSGL-1, which had been prelabeled with Zenon AlexaFluor594. We observed that the uropod containing Gag-YFP maintained contact with CMAC-stained T cells for over 20 min as the cells moved through the field (Figure 3A and Movie S2). These observations suggest that HIV-1-infected T cells are able to mediate stable contacts with target cells through their uropods. We next quantified the newly formed contacts between Gag-YFP-expressing primary T cells and CMAC-stained target primary T cells formed during a 3-h coculture period. An example of T cell contacts is shown in Figure 3B. We found that the majority of newly formed contacts occurs at the uropod (Figure 3C), despite the average uropod constituting only approximately 25% of the total cell surface (data not shown). These results indicate that Gag-containing uropods stably and preferentially form new contacts with uninfected T cells. In these experiments, when target cells are also polarized, infected cell uropods formed a similar number of contacts with both ends of target cells (data not shown).

### Gag-containing uropods of infected cells participate in the formation and/or structure of the VS to which CD4 of target T cells accumulates

**Figure 4:**
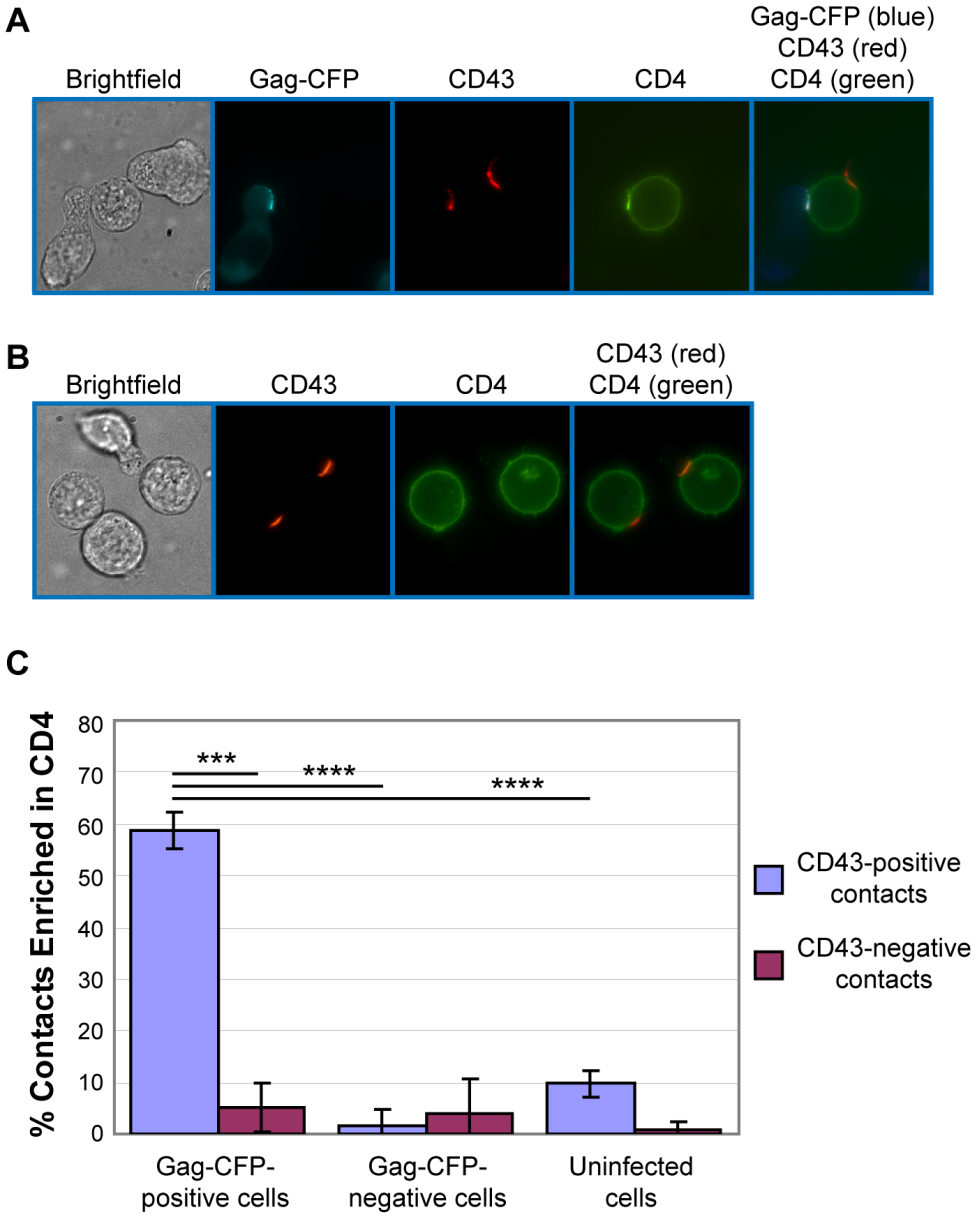
Gag-positive uropods form contacts enriched in CD4. **A**) P2 cells expressing Gag-CFP (cyan; pseudo-colored in blue in the merge panel) were immunostained with anti-CD43 and AlexaFluor-594-conjugated anti-mouse IgG (red). Subsequently, these cells were cocultured with SupT1 cells prelabeled with FITC-conjugated anti-CD4 (green) for 3 h and examined by live cell microscopy. A target cell (center) in contact with both Gag-CFP-positive (left) and Gag-CFP-negative (right) cells is shown. **B**) Uninfected P2 cells were immunostained with anti-CD43 and co-cultured with SupT1 cells prelabeled for CD4 as done in (A). **C**) In experiments represented in panels A and B, junctions between target SupT1 cells and Gag-CFP-expressing, non-Gag-CFP-expressing, or uninfected P2 cells were classified into uropod-mediated and non-uropod-mediated contacts based on the presence of CD43. The percentage of contacts with accumulation of CD4 relative to total contacts was determined for each category. Data from three independent experiments were shown as means +/−standard deviation. P values were determined using Student's t test. ***, P<0.001; ****, P<0.0001. Numbers of contacts detected and examined for CD4 accumulation in each of these three experiments are: Gag-CFP-positive/CD43-positve contacts, 56, 56, 57 (total 169); Gag-CFP-positive/CD43-negative contacts, 21,17,18 (total 56); Gag-CFP-negative/CD43-positive contacts, 26, 35, 39 (total 100); Gag-CFP-negative/CD43-negative contacts, 17, 24, 28 (total 69); uninfected/CD43-positve contacts, 56, 67, 86 (total 209); and uninfected/CD43-negative contacts, 20, 38, 55 (total 113).

It is possible that cell contacts formed by the infected cell uropods observed above actively participate in VS formation. To address this possibility, we examined localization of CD4, which is known to accumulate to the VS on the cell surface of target cells. P2 cells infected with VSV-G-pseudotyped HIV-1 expressing Gag-CFP and Env were immunostained for CD43 and mixed with target cells prelabeled with non-blocking, FITC-conjugated anti-CD4. After 3 h of coculture, cells were analyzed by live cell microscopy. When infected P2 cells were in contact with target SupT1 cells, CD4 on the surface of SupT1 cells accumulated to junctions formed between Gag-CFP-positive, CD43-positive uropods and target cells (Figure 4A). We found during quantitative analyses that CD4 accumulation to the cell-cell junctions predominantly takes place when Gag-CFP-positive uropods, but not non-uropod regions, of infected P2 cells are in contact with target SupT1 cells (Figure 4C). Such CD4 accumulation was rarely observed at junctions formed between Gag-CFP-negative or uninfected P2 cells and SupT1 cells (Figure 4A–C). These results suggest that infected T cell uropods are actively involved in recruitment of CD4 to cell junctions, perhaps through accumulation of Env (Figure 2). As cell junctions enriched in viral antigens such as Gag and the HIV receptor CD4 are defined as the VS in previous studies [61], these results support a model in which uropods or uropod-derived membrane components specifically participate in formation of the VS.

### Myosin light chain kinase inhibitor depolarizes cell morphology and Gag localization and reduces cell-to-cell transfer of Gag-YFP

**Figure 5:**
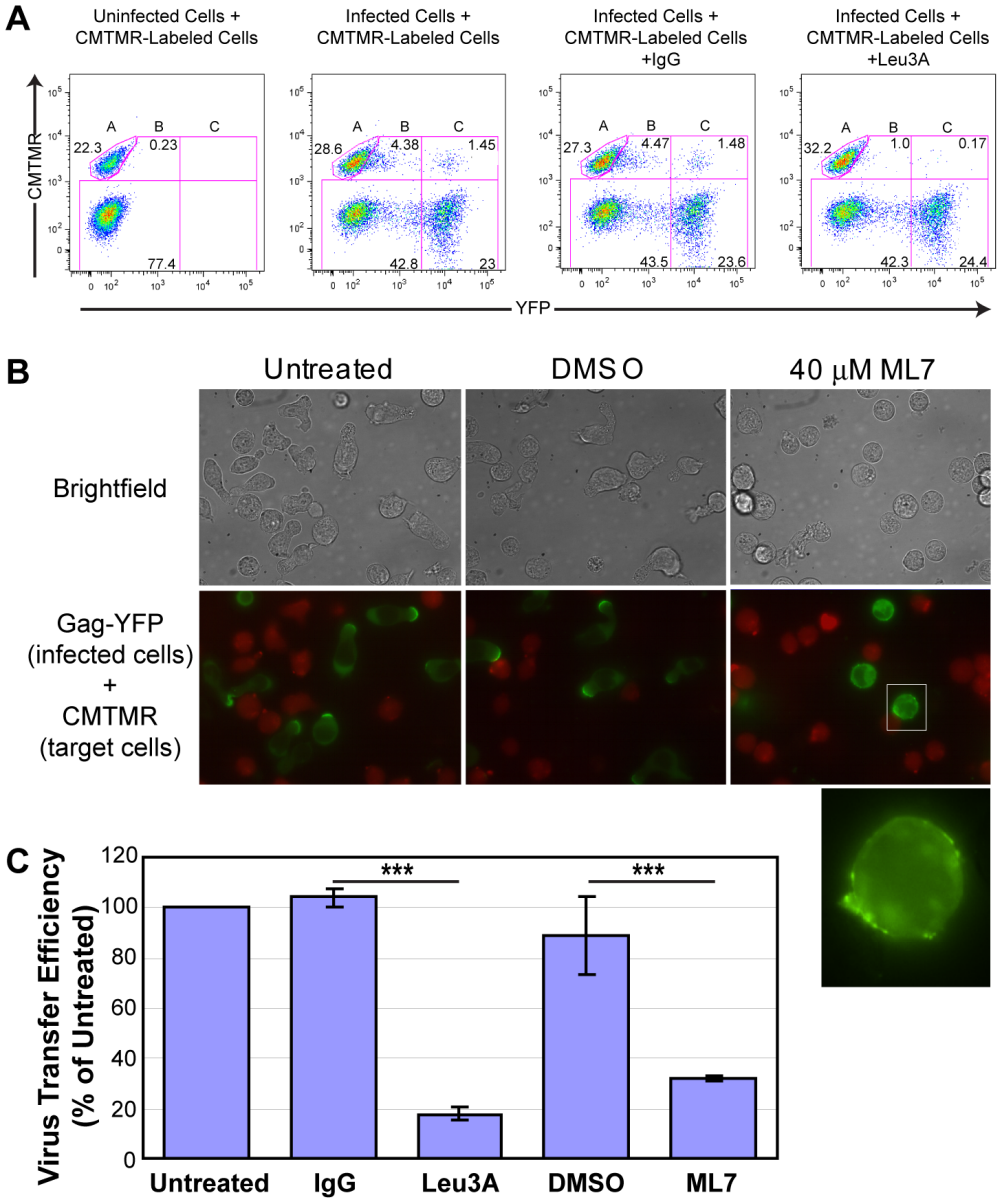
ML7 depolarizes cell morphology and Gag localization and reduces cell-to-cell transfer of Gag-YFP. **A**) Transfer of Gag-YFP fluorescence from infected P2 cells to CMTMR-stained SupT1 target cells during a 3-h coculture period was measured by flow cytometry. ML7, DMSO, or antibodies, along with 10 µg/ml cycloheximide, were added at the beginning of the coculture period. Flow cytometry plots for CMTMR-labeled target cells co-cultured with uninfected cells as well as CMTMR-labeled target cells cocultured with Gag-YFP-expressing infected cells in the presence or absence of IgG or Leu3A are shown. Gate A, CMTMR-labeled target cells; gate B, double positive cells representing target cells with transferred Gag-YFP particles; and gate C, YFP-expressing cells either fused or conjugated to CMTMR-labeled target cells. **B**) Images of cycloheximide-treated P2 cells expressing Gag-YFP were acquired after 3-h coculture with CMTMR-stained SupT1 cells in the presence or absence of DMSO or ML7. Note that almost all the cells adopt round unpolarized morphology upon treatment with ML7 and that ML7-treated infected cells show dispersed Gag-YFP localization. The latter point is clearer in the higher magnification image (bottom panel) of a region specified in the middle row. Also note that the cell density of the cocultures in experiments shown in panel A (images shown in Figure S3 and discussed in Text S1) is 10 fold higher than in panel B. **C**) Relative efficiencies of cell-to-cell virus transfer were calculated as the percentage of double positive cells out of the total CMTMR-labeled cells (Virus transfer efficiency = B/(A+B+C)*100; error bars represent standard deviation). P values were determined using Student's t test. ***, P<0.001.

In order to explore whether Gag accumulation to uropods facilitates transmission of HIV-1, we performed a cell-to-cell virus transfer assay. In this flow-cytometry-based assay, we measured transfer of YFP fluorescence, representing virions, from infected P2 cells expressing Gag-YFP to CMTMR-stained SupT1 target cells. Similar assays have been used in previous studies for analyzing cell-to-cell virus spread [53], [71], [102], [103], [104]. Representative flow cytometry plots for control cocultures are shown in Figure 5A. In these assays, we and others have observed that binding of cell-free virions to target cells is undetectable [53] (data not shown). Therefore, transfer of fluorescence represents cell-to-cell virus transfer. Consistent with previous reports [53], we observed a significant decrease in virus transfer when cells were cocultured in the presence of an anti-CD4 antibody (Leu3A) that prevents CD4-Env interaction, but not an isotype control IgG (Figure 5A and C). These data confirm the importance of Env in cell-to-cell transfer of HIV-1. Using this assay, we examined effects of cell depolarization on cell-to-cell HIV-1 transfer using a myosin light chain kinase inhibitor, ML7. As expected, treatment of Gag-YFP-expressing P2 cells with this inhibitor disrupted uropod formation and dispersed Gag-YFP on the plasma membrane (Figure 5B). ML7 did not have a major impact on efficiency of VLP release by Gag-YFP calculated as the amount of virion-associated Gag as a fraction of total Gag (Figure S2 and Text S1). However, because ML7 treatment reduces protein synthesis (data not shown), it was possible that any decrease in cell-to-cell virus transfer by treatment with ML7 may have arisen from reduced Gag expression instead of disruption of cell polarity. To rule out the indirect effect of protein synthesis inhibition on virus transfer, we included 10 µg/ml cycloheximide in all coculture conditions, including those treated with Leu3A and control IgG described above. As shown in Figure 5A, substantial virus transfer occurred even in the presence of cycloheximide. Finally, we observed that in the presence of cycloheximide, ML7 treatment significantly decreased cell-to-cell virus transfer (Figures 5C and S3). Together with the data showing that the uropod participates in formation of the VS (Figure 4), these results suggest that polarized localization of Gag and/or assembling particles at the uropod contributes to cell-to-cell transfer of virus particles to target cells.

### Gag localizes to uropod-specific microdomains

**Figure 6:**
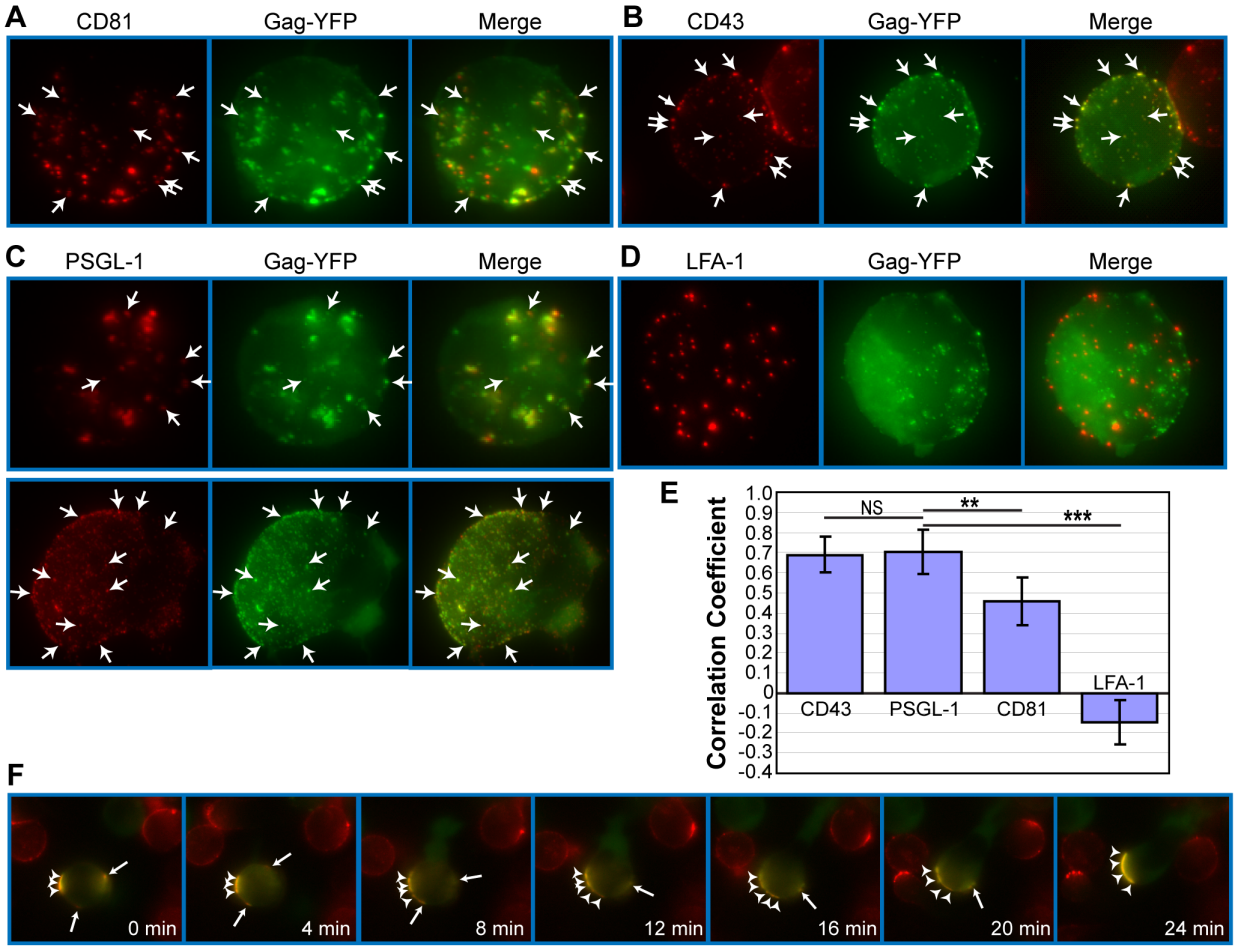
Gag associates with uropod-specific microdomains that carry Gag to the uropod. Unpolarized P2 cells expressing Gag-YFP (green) were examined for copatching with CD81 (**A**), CD43 (**B**), PSGL-1 (**C**), or LFA-1 (**D**). Cells were incubated with specific primary mouse monoclonal antibodies premixed with the fluorescent secondary antibody (red) followed by fixation. Z series of images of morphologically unpolarized cells were acquired and used to generate maximum projection images of each color using Metamorph 6 software. Merged images are shown in the right column of each panel. Several small copatching puncta are indicated by arrows. **E**) Quantification of copatching. Correlation coefficients between Gag-YFP and cell surface marker signals were calculated from a total of 18 cells for each marker. A value of 1 represents perfect colocalization, a value of -1 represents complete segregation, and a value of 0 represents a random distribution. P values were determined using Student's t test. NS, not significant. **, P<0.01. ***, P<0.001. **F**) Time lapse images of a Gag-YFP-expressing T cell during repolarization. Gag-YFP(green)-expressing P2 cells were immunostained for PSGL-1 (red) as described for panels A–D. Cells were then depolarized by incubation at 4°C for 30 min. Approximately 5 min after chamber coverslips containing depolarized cells were transferred to the microscope stage maintained at 37°C, acquisition of live cell images at indicated time points was begun. Note that the small patches (arrows) migrate and coalesce to the large patch (arrowheads) at the cell pole that eventually forms the uropod.

Because the results presented thus far suggest that Gag-laden uropods play a major role in cell-to-cell virus transmission, we next sought to elucidate the mechanism by which Gag accumulates to uropods. Gag has been shown previously to associate with microdomains, such as lipid rafts and tetraspanin-enriched microdomains (TEMs) [10], [54], [61], [105], [106], [107], [108], [109], [110], [111], [112], and these microdomains are observed at the VS [61], [80], [113], [114]. Since subsets of these microdomains are implicated in polarized localization of proteins in leukocytes [49], [60], [115], [116], [117], [118], it is conceivable that Gag utilizes uropod-specific microdomains for transport to the uropod. In this case, one would expect to observe copartitioning of Gag and uropod markers to the same microdomain even in unpolarized cells. A common method to test whether two proteins share a propensity for associating with the same microdomain is to test for colocalization, or “copatching”, after crosslinking with antibodies specific to each of the two proteins [119], [120], [121], [122], [123], [124]. We used this assay to examine whether Gag-YFP associates with uropod-directed microdomains in unpolarized P2 cells. As Gag forms multimers on its own, antibody-mediated crosslinking was needed only for cell surface marker proteins that include the uropod markers PSGL-1 and CD43 and the non-uropod marker LFA-1. Because Gag has been previously shown to colocalize with TEMs using similar methods [109], we also included the tetraspanin CD81 in the analysis. As observed in previous reports [109], we found that Gag copatches with CD81 (Figure 6A) (correlation coefficient or CC = 0.46; Figure 6E). Relative to CD81, however, the uropod markers PSGL-1 and CD43 copatched more extensively with Gag-YFP (CC = 0.69 and 0.70, respectively; Figure 6E). On the other hand, even though LFA-1 showed punctate localization as well, we observed a segregation of LFA-1 and Gag-YFP (Figure 6D) as indicated by the negative correlation coefficient (CC = −0.14; Figure 6E). Because copatching between Gag and uropod markers was observed even in non-polarized cells (Figure 6B and C), these results suggest that Gag localizes to uropod-specific microdomains prior to, and perhaps during, T cell polarization.

To examine whether Gag localized at uropods had originated from the Gag-positive patches observed in morphologically unpolarized cells, we conducted live-cell microscopy of Gag-YFP-expressing P2 cells that were first depolarized by low temperature treatment prior to image recording at 37°C. In these experiments, we observed that Gag-containing patches maintained colocalization with PSGL-1 and laterally moved on the plasma membrane to the forming uropod as cells re-polarized. (Figure 6F and Movie S3). Lateral movement of Gag-YFP was also observed in cells that were not immunostained for any marker, indicating that the observed movement was not caused by antibody-mediated crosslinking (Movie S4). These observations support a model in which Gag associates with uropod-specific microdomains while establishing localization at the rear end of polarized T cells.

### Env is not required for Gag localization to the uropod

**Figure 7:**
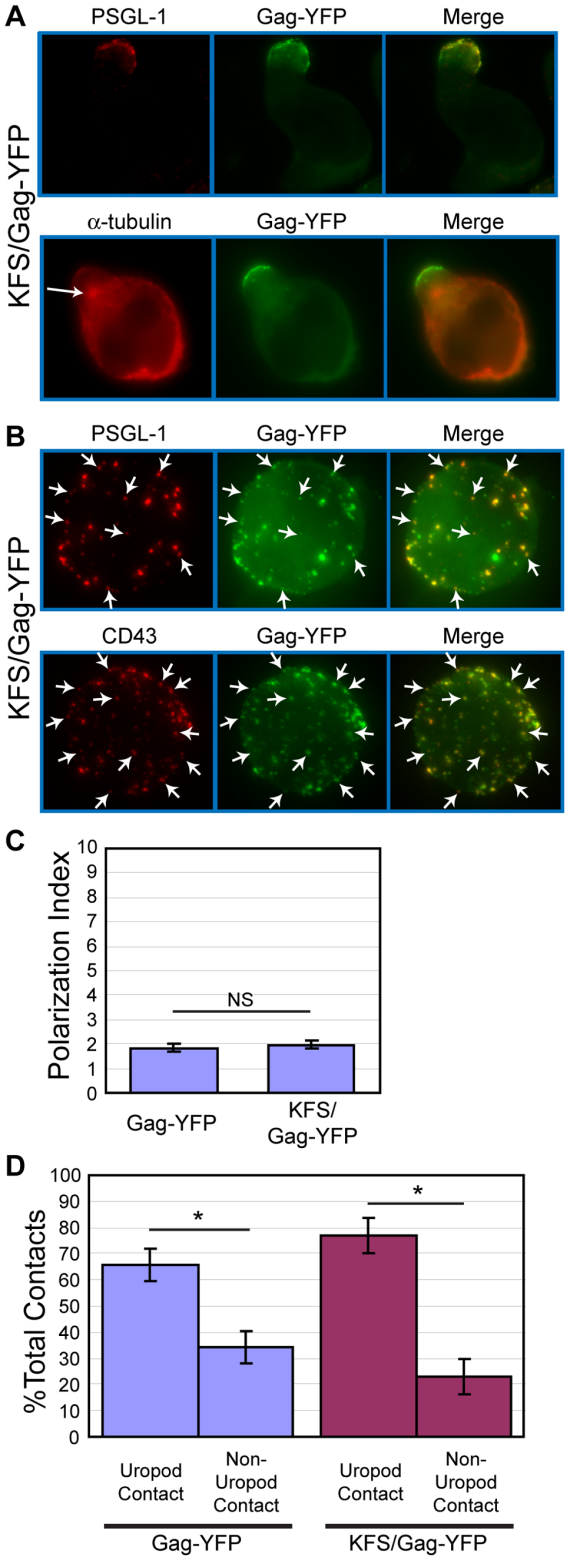
Env is not required for Gag localization to the uropod. **A**) P2 cells expressing KFS/Gag-YFP (green) were immunostained after fixation with anti-PSGL-1 (red, upper panel) or anti- α-tubulin (red, lower panel, arrow indicates MTOC) as described for Figure 1. **B**) KFS/Gag-YFP-expressing P2 cells were immunostained for PSGL-1 or CD43 (red) using the co-patching method as described for Figure 6, and Z-series of images of unpolarized cells were acquired. Maximum projections of each color were generated from the Z stacks and merged to examine colocalization (yellow). Several small copatching puncta are indicated by arrows. **C**) The Gag polarization index was determined as described in the Materials and Methods section for cells expressing Gag-YFP and KFS/Gag-YFP. Four separate experiments (32, 23, 39, and 37 cells each) for a total of 131 cells for Gag-YFP and 3 separate experiments (34, 48, and 30 cells each) for a total of 112 cells for KFS/Gag-YFP were used for quantification of Gag polarization. P values were determined using Student's t test. NS, not significant. **D**) Cell-cell contact assays were performed as in Figure 3. This graph compares the results of Figure 3 to the results obtained with cells expressing KFS/Gag-YFP, which had been obtained concurrently. P values were determined using Student's t test. *, P<0.05.

It has been shown that Gag and Env interact with each other [125], [126], [127], [128], [129], [130], [131]. Furthermore, it has been shown that Env is required for the formation of virological synapses between infected and uninfected T cells [53], [61], [67], [68], [78], [80], [89], unlike those formed between uninfected T cells and infected macrophages [75]. Therefore, Env may play an active role in Gag localization to uropods. To address this possibility, we examined T cells expressing an HIV-1 molecular clone that encodes Gag-YFP but not Env (KFS/Gag-YFP). In these cells, Gag-YFP co-localized strongly with PSGL-1 and copolarized with the MTOC at the uropod (Figure 7A), just as observed in cells expressing both Gag-YFP and Env (Figure 1). To examine microdomain partitioning, we also performed copatching assays for KFS/Gag-YFP and uropod markers in unpolarized cells. We observed that KFS/Gag-YFP copatches with the uropod markers PSGL-1 and CD43 (Figure 7B) at comparable levels to wild type (data not shown). We also compared Gag polarization indices between cells expressing Gag-YFP in the presence (Gag-YFP) or absence (KFS/Gag-YFP) of Env. The Gag polarization index describes the extent of Gag distribution along the plasma membrane from one cell pole to the other (see Materials and Methods). A lower index represents stronger polarization. We observed that the polarization index for KFS/Gag-YFP is nearly identical to that for Gag-YFP (Figure 7C, p = 0.28). We also found that the absence of Env had no impact on the preference for uropod-mediated contact between Gag-YFP-expressing primary T cells and CMAC-stained target primary T cells (Fig 7D, p = 0.23). This finding suggests that Env may not be required for initial contact formation, even while it is required for transfer of virus particles (Figure 5) [53], [132] and maintenance of cell-cell conjugates [53], [68], [78], [80]. Taken together, these results indicate that HIV-1 Env is dispensable for localization of Gag to the uropod.

### MA and CA are not required for Gag localization to the uropod

**Figure 8:**
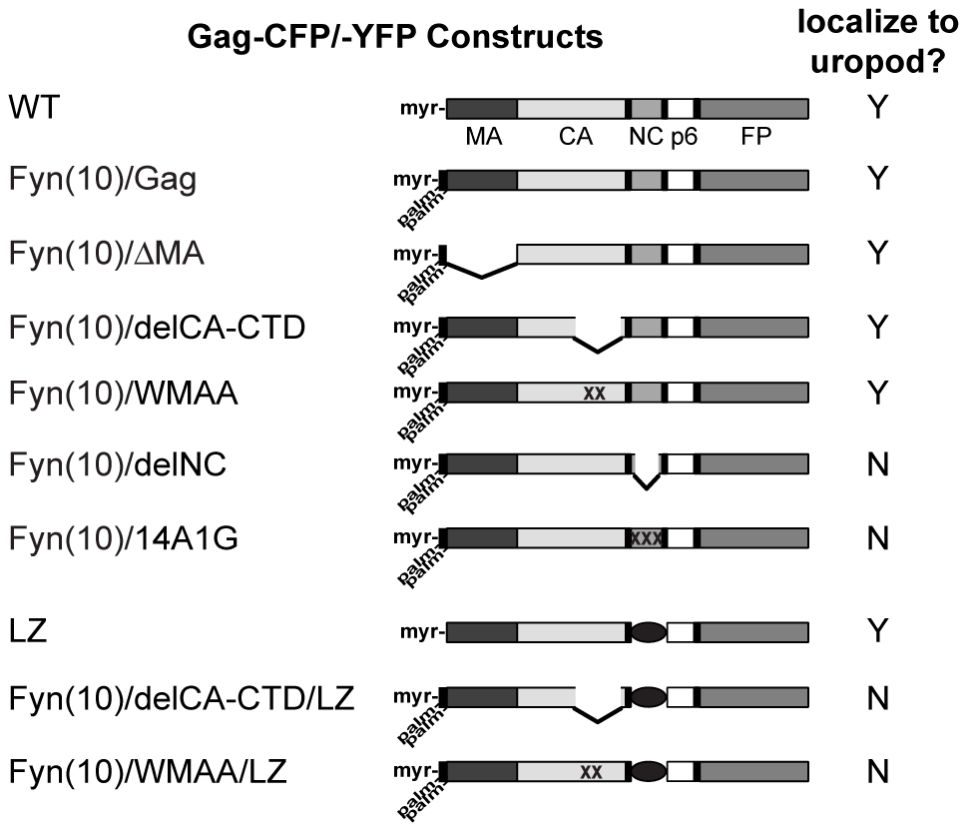
Gag derivatives used in this study and their localization pattern. HIV-1 molecular clones that express Gag-fluorescent protein fusions (WT Gag-YFP/-CFP) were generated. Deletions or amino acid substitutions were created in the MA domain (Fyn(10)/ΔMA/Gag-YFP/-CFP), CA domain (Fyn(10)/delCA-CTD/Gag-YFP/-CFP and Fyn(10)/WMAA/Gag-YFP/-CFP) and the NC domain (Fyn(10)/delNC/Gag-YFP/-CFP, Fyn(10)/14A1G/Gag-YFP/-CFP and LZ/Gag-YFP/-CFP). Gag derivatives containing LZ replacement of NC combined with the CA mutations were also used (Fyn(10)/delCA-CTD/LZ/Gag-YFP/-CFP and Fyn(10)/WMAA/LZ/Gag-YFP/-CFP). The Fyn(10) sequence, a single myristylation and dual palmitoylation signal, was added to the N terminus of all Gag derivatives except WT Gag-CFP/-YFP and LZ/Gag-CFP/-YFP. Results as to whether these Gag derivatives localize specifically to the uropod (Y) or distribute over the entire cell surface (N) are summarized in the right column.

**Figure 9:**
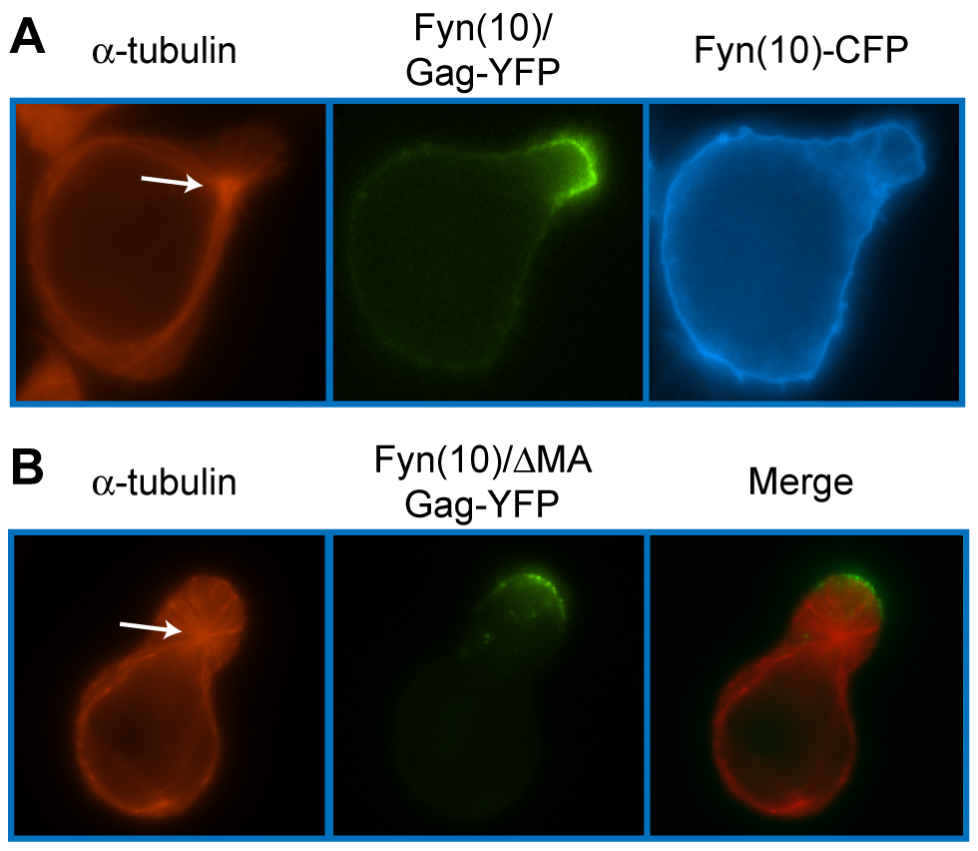
The MA sequence is not required for Gag localization to the uropod. **A**) P2 cells were co-transfected with plasmids that express Fyn(10)/Gag-YFP (green) and Fyn(10)-CFP (blue). Cells were then stained for α-tubulin (red, arrow indicates MTOC). **B**) Cells expressing Fyn(10)/ΔMA/Gag-YFP (green) were immunostained with anti-α-tubulin (red, arrow indicates MTOC).

To identify the molecular determinants of Gag that facilitate its localization to the uropod, we examined a panel of Gag mutants (Figure 8). Because MA is essential for specific targeting of Gag to the plasma membrane [11], [12], [100], [133], [134], [135], [136], [137], [138], [139], it is conceivable that MA also regulates specific localization of Gag to uropods. To test this possibility, we examined the effect of MA deletion on Gag localization to uropods. As MA is also essential for general membrane binding, to restore Gag membrane binding of the MA deletion mutant, we added to the N terminus of Gag a heterologous membrane binding sequence, an N-terminal 10-amino-acid sequence of Fyn kinase [Fyn(10)]. This sequence contains acylation signals for one myristoyl and two palimitoyl groups, and fully restores Gag membrane binding in the absence of the entire MA sequence [11]. Notably, the Fyn(10) sequence by itself is not capable of targeting proteins to uropods. As shown in Figure 9A, CFP attached to the Fyn(10) sequence [Fyn(10)-CFP] localized around the entire plasma membrane. In contrast, Fyn(10)/Gag-YFP localized to the uropod in the same cell (Figure 9A). These results indicate that the addition of Fyn(10) did not alter uropod localization of full-length Gag [Fyn(10)/Gag-YFP] in T cells, and that some region in Gag is required for its uropod localization. Notably, we observed that Fyn(10)/ΔMA/Gag-YFP, in which the entire MA sequence is deleted, still localized to the uropod efficiently in T cells (Figure 9B). Taken together, these results indicate that Gag localization to the uropod requires sequences downstream of MA and not the MA sequence itself.

**Figure 10:**
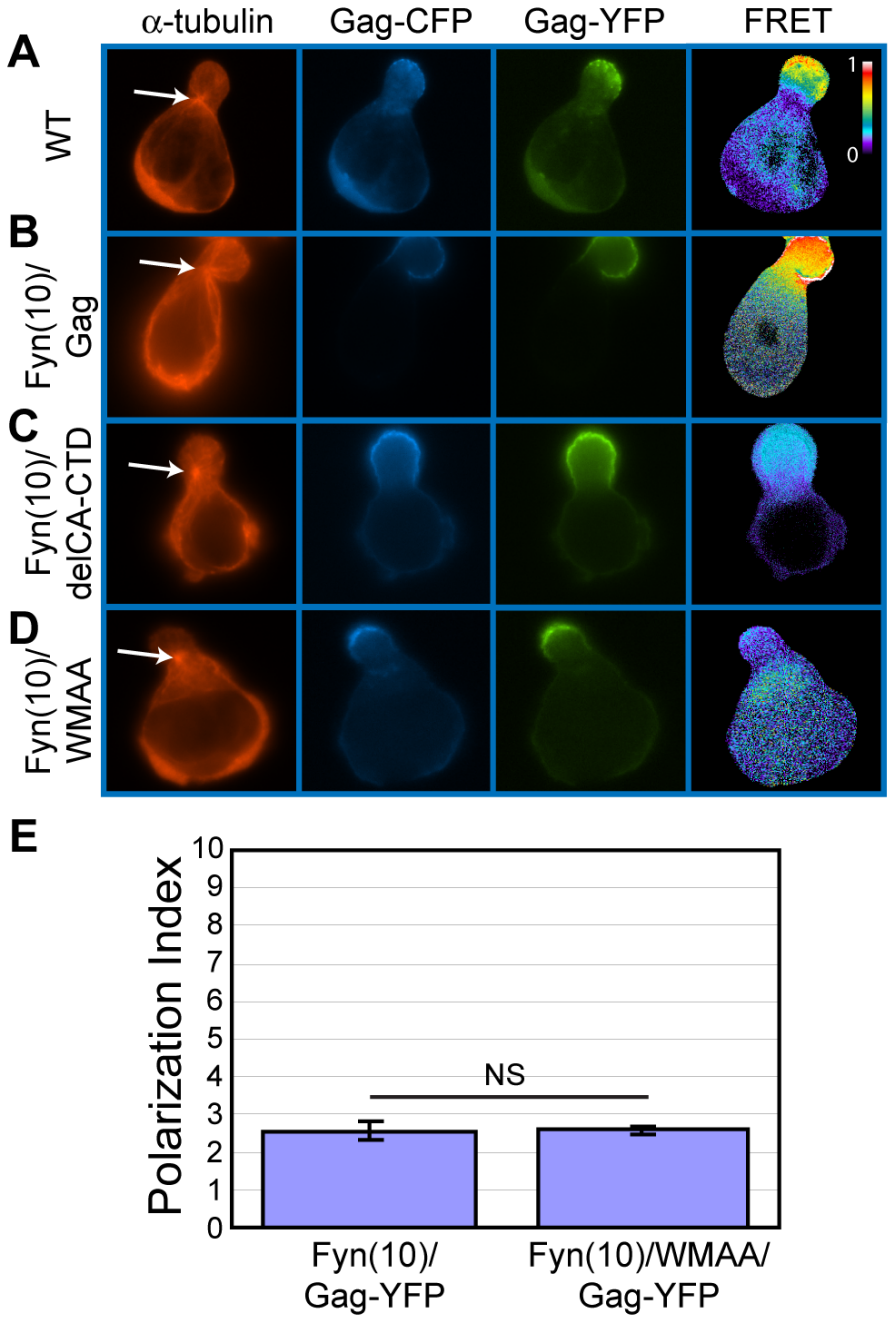
CA-mediated dimerization is not required for Gag localization to the uropod. **A-D**) P2 cells expressing Gag-YFP (green) and Gag-CFP (blue) or their derivatives were stained for α-tubulin (red) to identify the MTOC. Multimerization efficiency of each Gag mutant was measured by FRET. **A**) WT Gag-YFP/-CFP, **B**) Fyn(10)/Gag-YFP/-CFP, **C**) Fyn(10)/delCA-CTD/Gag-YFP/-CFP, and **D**) Fyn(10)/WMAA/Gag-YFP/CFP. Note that Gag derivatives with CA changes both localize to the uropod but show low FRET. Color scale bar indicates colors associated with high (1) or low (0) FRET. **E**) Polarization indices were calculated for Fyn(10)/Gag-YFP and Fyn(10)/WMAA/Gag-YFP (see Materials and Methods). Three separate experiments (a total of 89 P2 cells for Fyn(10)/Gag-YFP and 78 P2 cells for Fyn(10)/WMAA/Gag-YFP) were used for quantification. Error bars represent standard deviation. P values were determined using Student's t test. NS, not significant.

The downstream sequence of MA includes CA and NC domains. During virus particle formation, these domains are known to promote the dimerization and multimerization of Gag. To examine the roles played by Gag-Gag interactions in uropod localization, we analyzed Gag derivatives with changes in either CA or NC. Because Gag multimerization defects also reduce steady-state membrane binding [28], [42], [140], the Fyn(10) sequence was added to the CA and NC mutants. We first examined the plasma membrane localization of two YFP- and CFP-tagged CA mutants: an amino acid substitution mutant WM184,185AA (Fyn(10)/WMAA/Gag-YFP/-CFP) and a deletion mutant lacking the C-terminal domain (Fyn(10)/delCA-CTD/Gag-YFP/-CFP). We observed previously by FRET microscopy that these CA mutants are deficient in Gag-Gag interactions in HeLa cells [28]. P2 cells were coinfected with VSV-G-pseudotyped viruses encoding derivatives of Gag-YFP or Gag-CFP, and their localization and multimerization were examined by fluorescence and FRET microscopy, respectively. WT Gag-YFP/-CFP and Fyn(10)/Gag-YFP/-CFP showed high FRET in the uropod, indicating that Gag multimers localize to uropods (Figure 10A and B). Notably, both Fyn(10)/delCA-CTD/Gag-YFP/-CFP and Fyn(10)/WMAA/Gag-YFP/-CFP also showed clear localization to the uropod (Figure 10C and D) although, as expected, these Gag mutants displayed low FRET (Figure 10C and D). The polarization index for Fyn(10)/WMAA/Gag-YFP was also nearly identical to that of Fyn(10)/Gag-YFP (Figure 10E). Taken together, these results demonstrate that CA-mediated dimerization is not required for localization of Gag to the uropod.

### NC is essential for localization of Gag to the uropod

**Figure 11:**
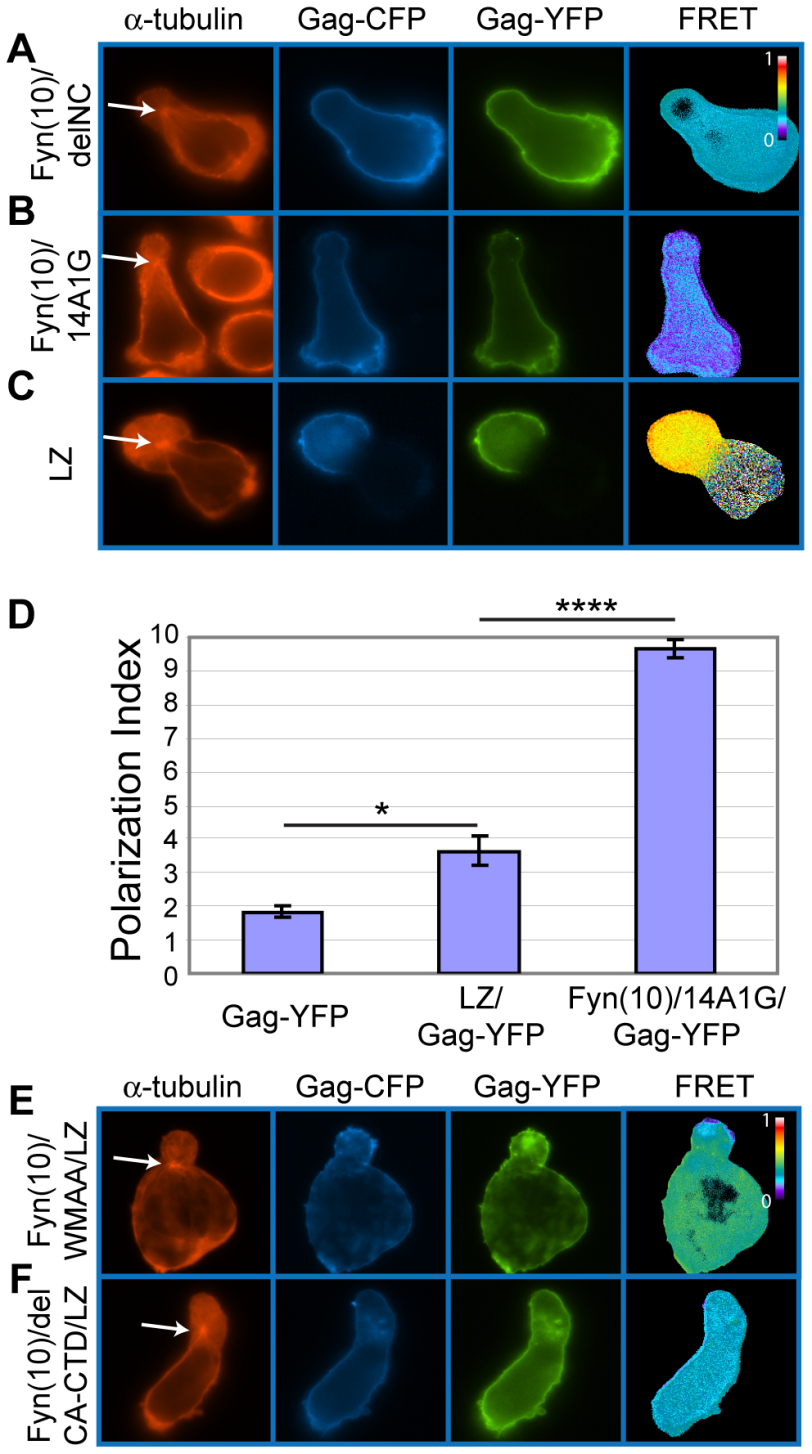
Higher-order multimerization mediated by NC is required for Gag localization to the uropod. **A–C**) P2 cells expressing Gag-YFP and Gag-CFP that contain an NC deletion or substitutions (green/blue) were immunostained with anti-α-tubulin (red, arrow indicates MTOC). Note that Fyn(10)/delNC/Gag-YFP/-CFP (**A**) and Fyn(10)/14A1G/Gag-YFP/-CFP (**B**) localize over the entire plasma membrane. Both mutants show reduced levels of FRET. In contrast, LZ/Gag-YFP/-CFP (**C**) mostly localizes to the uropod. High FRET is observed, indicating that multimerization is rescued as well. **D**) Polarization indices were calculated for LZ/Gag-YFP and Fyn(10)/14A1G/Gag-YFP and compared to WT Gag-YFP. LZ/Gag-YFP is less efficient in polarization, but significantly more efficient than Fyn(10)/14A1G/Gag-YFP. A total of 131 cells for Gag-YFP, 79 cells for LZ/Gag-YFP, and 69 cells for Fyn(10)/14A1G/Gag-YFP from three separate experiments were measured for polarized localization of Gag. Error bars represent standard deviation. P values were determined using Student's t test. *, P<0.05. ****, P<0.0001. **E**) and **F**) Double mutants containing the LZ sequence with the two different changes in the CA C-terminal domain, Fyn(10)/WMAA/LZ/Gag-YFP/-CFP (E) and Fyn(10)/delCA-CTD/LZ/Gag-YFP/-CFP (F), were expressed in P2 cells. Note that both Gag derivatives fail to localize to the uropod despite the presence of the LZ sequence.

To examine the role of NC in Gag localization to uropods, we next analyzed a mutant Gag that lacks most of the NC sequence (Fyn(10)/delNC/Gag-YFP/-CFP). In contrast to the MA and CA mutants that localized to the uropod, Fyn(10)/delNC/Gag-YFP/-CFP localized over the entire plasma membrane (Figure 11A). An NC mutant in which 15 NC basic residues essential for RNA binding were substituted with alanine or glycine (Fyn(10)/14A1G/Gag-YFP/-CFP) also showed non-polarized localization (Figure 11B). These results indicate that NC is required for Gag localization to the uropod. Pleitropic impacts of NC mutations on Gag assembly precluded us from obtaining interpretable results regarding the effects of these mutations on cell-to-cell transfer (Figure S2 and Text S1).

### NC-mediated multimerization is required for Gag localization to the uropod

As confirmed by FRET microscopy (Figure 11A and B), both Fyn(10)/delNC/Gag-YFP/-CFP and Fyn(10)/14A1G/Gag-YFP/-CFP that are defective in polarized localization are also defective in Gag-Gag interaction. Therefore, it is possible that NC-mediated Gag multimerization or clustering plays a key role in Gag localization to the uropod. Alternatively, other functions of NC may facilitate Gag localization to the uropod. To distinguish between these possibilities, we examined a Gag derivative in which NC was replaced by a leucine zipper sequence (LZ) derived from GCN4 (LZ/Gag-YFP/-CFP). Gag derivatives in which NC is replaced with this LZ sequence, which has no homology to NC, have been shown previously to multimerize efficiently [141], [142], [143]. We observed that LZ/Gag-YFP/-CFP localized to the uropod in a majority of cells expressing this Gag derivative and yielded a WT level of FRET (compare Figure 11C with Figure 10A). Quantitative analysis of polarization indicated that LZ/Gag-YFP was not as efficiently polarized as WT, but nonetheless significantly more polarized than the NC point mutant Fyn(10)/14A1G/Gag-YFP (Figure 11D). These results suggest that NC promotes Gag localization to the uropod through its ability to facilitate higher-order Gag multimerization. As the LZ sequence used above is a dimerization sequence, it would drive higher-order multimerization only in the presence of an additional dimerization motif such as CA-CTD. Thus, we hypothesized that although Fyn(10)/delCA-CTD/Gag-YFP/-CFP and Fyn(10)/WMAA/Gag-YFP/-CFP localize to uropods (Figure 10C and D), in these contexts, LZ in the place of NC would be unable to promote Gag localization to uropods (Figure 11E and F). Indeed, cells expressing these constructs, Fyn(10)/WMAA/LZ/Gag-YFP and Fyn(10)/delCA-CTD/LZ/Gag-YFP, showed localization of Gag over the entire plasma membrane, a pattern identical to that of the NC mutants (compare Figure 11E and F with A and B). Taken together, these results demonstrate that dimerization mediated by either CA-CTD or LZ alone is insufficient for localization to the uropod. However, NC-mediated higher-order multimerization or clustering of Gag, which likely occurs even in the absence of the CA-CTD dimerization motif, is essential for localization to the uropod.

## Discussion

In lymphoid organs, where HIV-1 likely spreads efficiently from infected to uninfected T cells, T cells adopt a polarized morphology and are highly motile. Thus, studying HIV-1 replication in polarized T cells may help us to better understand how the virus spreads *in vivo*. In this study, we found that HIV-1 Gag accumulates to, and forms mature virions at, the uropod in polarized T cells (Figures 1 and 2). These observations led us to ask whether uropod localization of HIV-1 Gag plays a role in the spread of the virus. In uninfected T cells, uropods are enriched in adhesion molecules and known to mediate contact with other cells [49]. Therefore, polarized virus assembly at uropods could facilitate cell-to-cell transmission of HIV-1. Consistent with this possibility, live cell microscopy and quantitative cell-cell contact analyses showed that HIV-1-infected cells contact target cells preferentially through their uropods (Figure 3). Furthermore, a substantial majority of Gag- and CD4-positive cell-cell contacts, which define the VS [61], were observed where uropod-derived (CD43-positive), but not non-uropod (CD43-negative), regions of infected cells mediated contact with target cells (Figure 4). Consistent with these microscopy data, we observed that ML7, a myosin light chain kinase inhibitor that blocks the polarization of T cells and formation of uropods, both dispersed Gag over the cell surface and reduced cell-to-cell transfer of virus particles significantly (Figure 5). We note that ML7-sensitive, actin-myosin-based processes besides cell polarization may affect cell-to-cell virus transfer. However, taken together, these results indicate that uropods of polarized T cells play an important role in cell-to-cell transfer of HIV-1. Notably, bone marrow hematopoietic stem cells have been shown to mediate not only contacts with osteoblasts, but also cell-to-cell transfer of plasma-membrane-associated molecules via their uropods [144]. This process, postulated to mediate intercellular signal transfer, may be a common cell-cell communication mechanism shared among cells of the hematopoietic cell lineage, including T cells. Thus, localization of HIV-1 components to, and subsequent virus assembly at, the uropod may represent yet another example in which viruses hijack cellular processes to facilitate its own replication.

**Figure 12:**
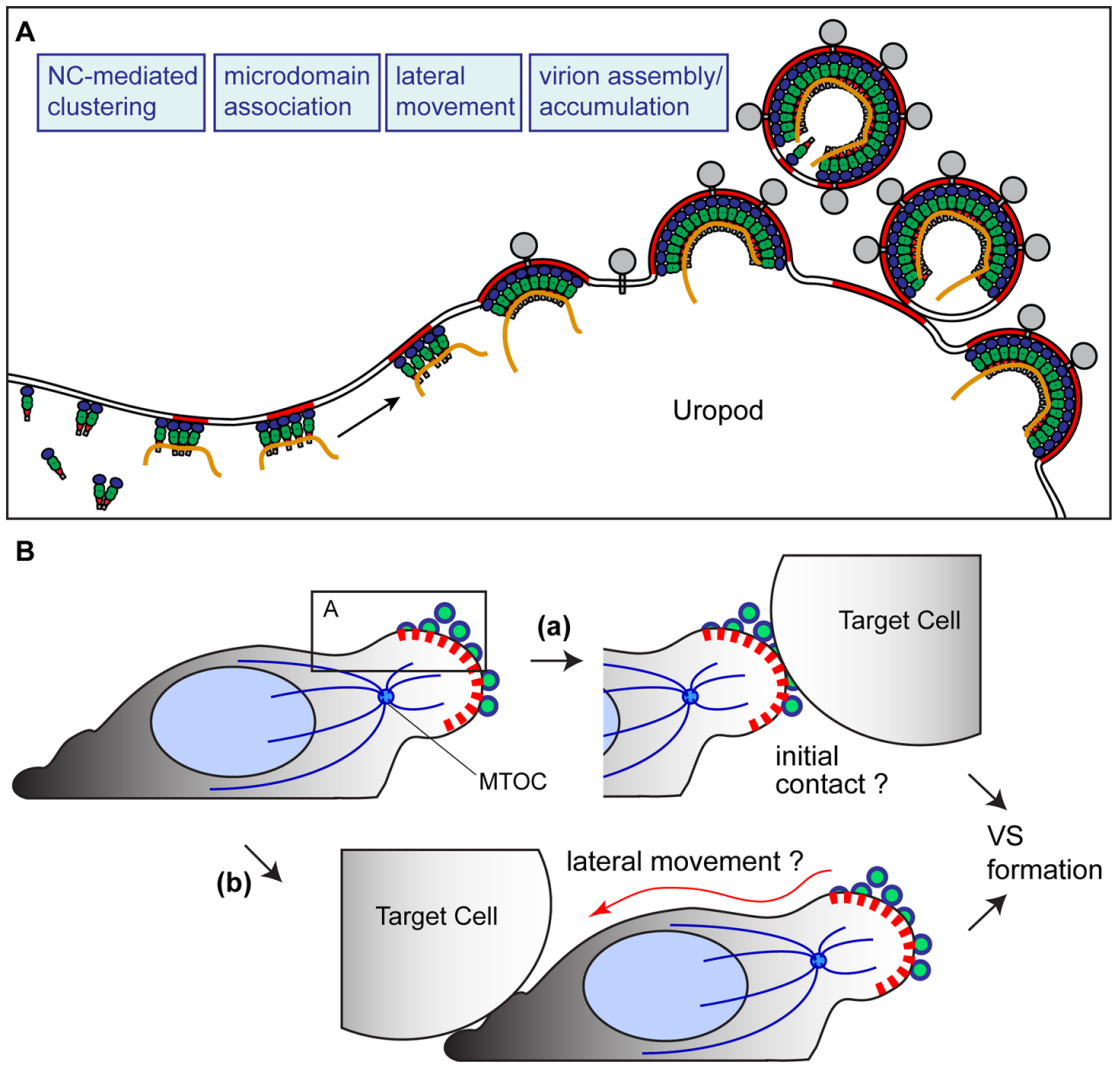
Working model. **A**) Based on the findings in this study, we have postulated a working model in which NC-mediated clustering of Gag allows association of Gag with uropod-specific microdomains that facilitate movement to the uropod in polarizing T cells. **B**) The virus-laden uropod, acting as a pre-formed platform that may either mediate contact with target cells (a) or relocalize to contacts formed elsewhere subsequently (b), constitutes a VS that facilitates cell-to-cell transmission of HIV-1.

It has been reported by several groups that cell-to-cell HIV-1 transmission occurs via the VS. However, the steps leading to formation of the VS are not well defined. Observations described in this study suggest that at least one path toward establishment of the VS is the accumulation of viral components and assembling virions to the uropod. Uropods could then serve as a pre-formed platform that constitutes a VS upon cell-cell contact [Figure 12B (a)]. Consistent with this possibility, previous studies showed that disruption of the cytoskeleton, which also impairs cell polarity, reduces Gag accumulation to contact sites between infected and uninfected T cells [77], [78], [80], [145]. It is conceivable that suppression of uropod formation or inhibition of Gag localization to uropods may account for the observed reduction of VS formation upon cytoskeleton disruption.

It is important to note that our data do not preclude other modes of VS formation. For example, if morphologically unpolarized cells with dispersed Gag make contact with a target cell, Gag could re-localize laterally to the contact site. Consistent with such Gag movement, a recent imaging study showed that most cell conjugate formation precedes Gag redistribution when apparently unpolarized Jurkat cells were used as donor cells [67]. Such lateral movement could also occur in polarized cells that initially contact target cells through a non-uropod region of the cell [Figure 12B (b)]. Movement of Gag-containing patches to contact sites has been observed in recent VS studies [67], [80]. It is possible that these patches may have originated at the uropod, although this point remains to be determined by long-term live cell monitoring of polarized T cells. Thus, in either mode of VS formation, prior formation of a platform enriched in Gag and other viral components, which takes place at the uropod, may be an important first step in cell-to-cell virus transfer.

Our data support that polarized localization of Gag to the uropod plays an important role in HIV-1 spread. If so, what drives localization of Gag and virus assembly to uropods? Previous studies have shown that some cell-surface proteins localize to the uropod upon antibody crosslinking through an undefined mechanism [146], [147], [148]. As dimerization and multimerization can be considered to be a form of crosslinking, we examined whether Gag localization to uropods similarly depends on Gag multimerization. While CA dimerization mutations did not alter localization of Gag to the uropod (Figure 10), mutations that disrupt higher-order multimerization mediated by NC-RNA interactions did (Figure 11). Mutations in NC caused Gag to localize over the entire plasma membrane despite the presence of the CA dimerization interface. Furthermore, a heterologous dimerization sequence, LZ, restored the uropod localization of NC-deleted Gag. Finally, this LZ-dependent localization required the intact CA dimerization interface, supporting the importance of higher-order Gag multimerization. Therefore, although both CA dimerization and NC-RNA interaction are important for Gag assembly, it is the NC-dependent higher-order multimerization that is essential for Gag localization to the uropod. In this regard, uropod localization of Gag may be driven by a mechanism similar to the one targeting multimerizing proteins to endosome-like domains reported recently to exist on the plasma membrane[149]. The nature of the NC-dependent higher-order multimer directed to uropods remains to be elucidated; however, as CA dimerization mutants that did not yield substantial FRET signals still localized to uropods (Figure 10), it is likely that the uropod targeting process does not require the NC-dependent multimer to be in a highly aligned and packed form. As NC by itself can bind RNA in the absence of CA [150], we speculate that Gag clustering through binding to the same RNA molecule is sufficient for localization to uropods.

Protein-protein interactions, which include clustering or multimerization of membrane proteins, are known to stabilize the microdomains with which those proteins associate [151]. In this study, we showed that Gag copatches moderately with CD81 and strongly with uropod markers PSGL-1 and CD43 even in non-polarized cells (Figure 6). We also observed, using live cell analysis, that Gag patches move laterally on the cell surface of unpolarized cells and accumulate at the forming uropod as cells polarize. These results support a model in which Gag, a multimerizing protein, associates with uropod-specific microdomains that carry Gag to the uropod. However, the mechanism by which these microdomains localize to the uropod remains unclear. It is important to note that not all types of microdomains are destined for the uropod. GM3-containing lipid rafts have been shown to localize to the leading edge [60], [118], [152]. Therefore, it is likely that there are complex sorting mechanisms by which specific subsets of microdomains are moved to the uropod. In this regard, it is of note that although LFA-1 behaves as a leading edge/non-uropod marker in T cells in suspension [153] (this study), this adhesion molecule redistributes to mid-cell and uropod regions upon contact with ICAM-1-containing surfaces [154], [155]. Therefore, LFA-1 in infected cells may still modulate uropod-mediated T-cell-T-cell contacts upon encountering ICAM-1-bearing target cells, which would be in agreement with previous studies [96], [103].

While our data showed that patches of Gag laterally move to the uropod as cells re-polarize, they do not rule out the possibility that in an already polarized cell, *de novo* assembly of viruses preferentially occurs at the uropod or the cell contact without the lateral movement of Gag clusters. A recent study showed that MLV, another retrovirus, preferentially forms particles at contact sites in HEK293 cells [88]. This observation indicates that the site of retrovirus assembly can be polarized upon cell-cell contact formation in otherwise unpolarized cells. Notably, the polarized budding of MLV in HEK293 cells was found to be dependent on the MLV Env cytoplasmic tail. Similarly, the cytoplasmic tail of HIV-1 Env was reported to be important for polarized HIV-1 Gag localization in Jurkat T cells that appeared morphologically unpolarized [156]. In contrast, in our study, we found that in the absence of Env or cell-cell contact, Gag-YFP remained efficiently localized to the uropod in polarized T cells, including P2 and primary CD4^+^ T cells (Figures 1G and 7; data not shown). Therefore, it is possible that in T cells with a high propensity to establish front-rear polarity, Gag may not require Env or cell-cell contact to achieve polarized assembly. Further studies will determine the molecular mechanisms by which assembly sites for retroviruses are polarized in different cell types.

Although Env was dispensable for Gag localization to the uropod, formation of stable cell conjugates as well as virus transfer have been shown to require Env-receptor interaction [53], [67], [68], [78], [80], [132]. Consistent with these findings, we observed that anti-CD4 blocking antibody (Leu3A) diminished cell-to-cell virus transfer (Fig. 5) and that prelabeling of infected P2 cells with anti-Env antibody (b12) reduced formation of cell conjugates with SupT1 cells (data not shown). Therefore, while uropods are enriched in adhesion molecules and form contacts with other cells frequently [49] regardless of the presence of Env, the Env-CD4 interaction is likely to stabilize such contacts during formation of the VS.

In summary, this study elucidates a series of molecular events leading to the formation of a VS. The observations made in this study has led us to form a working model (Figure 12) in which higher-order multimerization, or clustering, mediated by NC is required for Gag association with uropod-specific microdomains. This microdomain association then facilitates localization of the assembling virus to the uropod. According to this model, the uropod, laden with HIV-1 components and particles, then serves as a pre-formed platform that mediates contact with target cells [Figure 12B (a)] or redistributes to contacts formed elsewhere [Figure 12B (b)]. Such contacts could then constitute a VS, which likely facilitates cell-to-cell virus transfer of HIV-1.

## Materials and Methods

### Plasmids

The HIV-1 molecular clone pNL4-3 [157] and its derivatives encoding Gag-YFP and Gag-CFP fusion proteins (pNL4-3/Gag-YFP/-CFP) [11], [28] were described previously. The latter two constructs contain an extensive deletion of *pol* and silent mutations to reduce ribosomal frameshift to the *pol* reading frame and does not express Vif or Vpr. For YFP and CFP, monomeric Venus [158], [159] and monomeric Cerulean [160] variants were used, respectively. pNL4-3/WM184,185AA/Gag-YFP/-CFP (renamed as pNL4-3/WMAA/Gag-YFP/-CFP), pNL4-3/delCA-CTD/Gag-YFP/-CFP, pNL4-3/14A1G/Gag-YFP/-CFP, pNL4-3/delNC/Gag-YFP/-CFP and the Fyn(10)-modified versions of those plasmids were previously described [28]. In this study, pNL4-3/Fyn(10)fullMA/GagVenus described previously [11], [28] was renamed as pNL4-3/Fyn(10)/Gag-YFP for simplicity. pNL4-3/Fyn(10)/ΔMA/Gag-YFP was previously described [11]. pNL4-3/KFS/Gag-YFP was generated by cloning the XhoI-SalI fragment of pNL4-3/KFS (a kind gift from Dr. Eric Freed [161]) into pNL4-3/Gag-YFP. To construct pNL4-3/LZ/Gag-YFP/-CFP, the sequence encoding GCN4 leucine zipper in the ZWT plasmid, a kind gift from Dr. Heinrich Gottlinger [142], was cloned into pNL4-3/Gag-YFP/-CFP using standard molecular cloning techniques. The double mutants pNL4-3/Fyn(10)/WMAA/LZ/Gag-YFP/-CFP and pNL4-3/Fyn(10)/delCA-CTD/LZ/Gag-YFP/-CFP were generated by cloning a fragment containing the leucine zipper sequence from pNL4-3/LZ/Gag-YFP into pNL4-3/Fyn(10)/WMAA/Gag-YFP/-CFP and pNL4-3/Fyn(10)/delCA-CTD/Gag-YFP/-CFP, respectively. pNL4-3/Gag-iGFP (a kind gift from Dr. Benjamin Chen [99]) was used to construct pNL4-3/Gag-iYFP.

### Virus stocks

Stocks of HIV-1 mutants, pseudotyped with vesicular stomatitis virus G protein (VSV-G), were prepared by transfecting 5.6×10^5^ 293T or HeLa cells with 1.5 µg pNL4-3 derivative encoding a Gag-YFP/-CFP fusion protein, 1.5 µg pCMVNLGagPol-RRE [105], and 0.5 µg pHCMV-G (a kind gift from Dr. J. Burns [162]). Two days post transfection, virus-containing supernatants were filtered through a 0.45 µm filter and used for inoculation of T cells.

### Cells

To prepare a polarized T cell line, T cell clones were obtained by limiting dilution of A3.01 T cells (AIDS Research and Reference Reagent Program). Typical A3.01 cell cultures naturally contain 10–20% of cells with a polarized morphology. After limiting dilution, T cell clones were examined for cell morphology and polarized PSGL-1 localization. A cell clone, in which approximately 50–60% of cells were polarized, was designated “P2” and used for experiments in this study. These cell lines, as well as the SupT1 cell line (AIDS Research and Reference Reagent Program), were cultured in RPMI containing 10% fetal bovine serum (FBS)(RPMI-10%FBS). Primary T cells were isolated from buffy coats obtained from the New York Blood Center. The buffy coats were diluted in a 1∶1 ratio with phosphate buffered saline (PBS) containing 2% FBS (PBS-2%FBS), and peripheral blood mononuclear cells (PBMCs) were isolated using centrifugation through ficoll (GE Healthcare) according to the manufacturer's instructions. Isolated PBMCs were then plated on polystyrene petri dishes for 2 h to separate the adherent monocytes and non-adherent lymphocytes. Lymphocytes were activated in RPMI-10%FBS containing phytohemagglutinin (PHA) (Sigma. St. Louis, MO) (6 µg/ml) and IL-2 (20 units/ml) (Roche. Basel, Switzerland) for 2–3 days. Primary CD4^+^ T cells were isolated with the MACS magnetic antibody bead kit (Miltenyi Biotec. Bergisch Gladbach, Germany) using anti-CD4 beads and MS columns. Cells were then cultured overnight in RPMI-10%FBS and IL-2 (20 units/ml) and used for experiments. IL-2 has been shown to induce a comparable level of T cell polarization and locomotion to those induced by chemokines such as RANTES and MIP-1α [163], [164].

### Infection

Cells were infected with virus stocks by spin infection; 3×10^5^ P2 cells or 5×10^5^ primary T cells were resuspended in 200 µl virus stock with 4 µg/ml polybrene and centrifuged at 2500 rpm for 2 h at 15°C. Cells were cultured at 37°C in RPMI-10% FBS for 2–3 days (in the presence of 20 units/ml IL-2 for primary T cells).

### Copatching assay

Mouse anti-PSGL-1 (NP_002997.1), CD43 (AH003828.1), CD81, or LFA-1 (all from BD Biosciences Pharmingen. San Diego, CA) were prelabeled with the secondary antibody (AlexaFluor-594-conjugated goat anti-mouse IgG (Invitrogen. Carlsbad, California)) for 30 min. Infected cells were cultured in 200 µl of RPMI-10%FBS containing this antibody mixture for 1 h at 37°C, after which they were washed with RPMI-10%FBS and fixed in 1 ml 4% paraformaldehyde in PBS (PFA). After washing with PBS-2%FBS, cells resuspended in a small volume (∼10 µl) of the same buffer were mixed with equal volume of Fluoromount-G (SouthernBiotech. Birmingham, Alabama), and 3 µl of this mixture was mounted on glass slides. Images were acquired with a Nikon TE-2000U inverted epifluorescence microscope. Z-series of images were acquired with 0.2 µm intervals between focal planes. Maximum intensity projection images of the z-series images composed of 56 focal planes were obtained with ImageJ software (NIH; downloaded from http://rsbweb.nih.gov/ij/). Copatching quantification was performed using the correlation plot function of the Metamorph 6 software (Molecular Devices. Sunnydale, California). To identify punctate signals objectively and to remove background signals from copatching analyses, the background, calculated as the median intensity of a 32×32-pixel region surrounding each pixel, was subtracted from the original image[165], point noise was removed using a 3×3 median filter[166], and the minimum threshold was set to twice the average fluorescence intensity of the cell of interest and applied to the images. These images were then used for calculation of Pearson's correlation coefficients (CC), representing copatching.

### Immunostaining

To avoid altering cell morphology, cell suspensions were placed in round-bottom tubes and left still at 37°C in the presence of 5% CO_2_ for at least 1 h prior to fixation. Subsequently, most of the culture supernatant was removed carefully, and cells were fixed in 1 ml 4% PFA for 20 minutes. Fixed cells were washed with PBS-2%FBS and then incubated for 1 h in PBS-2%FBS containing primary antibodies against cell surface molecules (PSGL-1 and LFA-1) followed by washing with PBS-2%FBS. For experiments in which CD43 was used as a uropod marker, cells were first incubated with anti-CD43 for 30 min as done in previous studies [64]. Subsequently, cells were rinsed with RPMI-10%FBS twice, incubated with AlexaFluor 594-conjugated anti-mouse IgG for 30 min, rinsed with RPMI-10%FBS twice, and cultured for an additional 30 min at 37°C prior to fixation. Detection of Env on the cell surface was performed similarly, except that primary and secondary antibodies used were anti-gp120 (IgG1 b12; AIDS Research and Reference Reagent Program) and AlexaFluor-594-conjugated anti-human IgG (Invitrogen), respectively. For detection of α-tubulin (to identify the MTOC) and mature p17MA, fixed cells were permeabilized by a 10-min incubation in PBS containing 0.2% saponin (Fluka Biohemica. Buchs, Switzerland) and 5% FBS prior to incubation with primary antibodies, anti-α-tubulin (Sigma; clone B-5-1-2) and anti-p17MA (Applied Biotechnologies. Columbia, Maryland), respectively. Primary antibodies were detected by treating cells with AlexaFluor 594-conjugated goat anti-mouse IgG for 30 minutes. Cells were then washed again with PBS-2%FBS and mounted on glass slides, as described above, for microscopy.

### Live cell microscopy

Cells were infected with VSV-G-pseudotyped HIV-1 encoding Gag-YFP. Two days post-infection, cells were immunostained with anti-PSGL1 prelabeled with Zenon AlexaFluor 594 reagent (Invitrogen) according to manufacturer's instruction or AlexaFluor 594-conjugated anti-mouse IgG as described for the copatching assay. To morphologically depolarize cells, 4-well chamber coverslips (Nunc. Rochester, NY), containing Gag-YFP-expressing cells, were placed at 4°C for 30 min. To repolarize cells, the chamber coverslips were then transferred to a pre-warmed (37°C) microscope stage. Time-lapse images were acquired with an interval of 30 s for up to 1 h. The images were then converted to AVI files by ImageJ.

### Fluorescence Resonance Energy Transfer (FRET) analysis

Cells were co-infected with VSV-G-pseudotyped HIV-1 encoding YFP- and CFP-tagged versions of each Gag mutant, cultured and fixed as described above. Cells were subsequently permeabilized, immunostained for α-tubulin, and mounted as described above. Images were collected using four filter combinations: AlexaFluor 594 excitation/AlexaFluor 594 emission, YFP excitation/YFP emission, CFP excitation/CFP emission, and CFP excitation/YFP emission. FRET was calculated using FRET stoichiometry as previously described [28], [167].

### Analysis of cell-cell contact and VS

5×10^5^ primary CD4^+^ T cells were infected with VSV-G pseudotyped HIV-1 encoding Gag-YFP or KFS/Gag-YFP. Two days post infection, 5×10^5^ fresh primary CD4^+^ T cells were stained with 1 µM CMAC (Invitrogen) for 30 min. Infected T cells were then co-cultured with CMAC-stained target T cells for 3 h in a chamber coverslip at 37°C. Images of 50–60 polarized and YFP-expressing cells were then acquired, and the number of contacts these cells formed with CMAC-labeled cells, which represent newly formed contacts, were quantified and categorized as uropod- or non-uropod-mediated contacts.

For analysis of the VS, 2×10^5^ P2 cells were infected with VSV-G-pseudotyped HIV-1 encoding Gag-CFP. Two days post-infection, cells were immunostained with anti-CD43 and a minimal amount of AlexaFluor-594-conjugated anti-mouse IgG. After extensive washing, 1×10^5^ of these cells or the same number of uninfected P2 cells were mixed with 1×10^5^ target cells (SupT1 cells) that were prelabeled with non-blocking FITC-conjugated anti-CD4 (Clone L120, BD Biosciences. San Jose, California) and cocultured for 3 h in chamber coverslips at 37°C. These cocultures in the chamber coverslips were placed on a microscope stage set at 37°C, and images were acquired using appropriate excitation and emission filters.

### Cell-to-cell virus transfer assay

2×10^5^ P2 cells were infected with VSV-G-pseudotyped HIV-1 encoding Gag-YFP. Two days post-infection, 5×10^5^ target SupT1 cells were stained with 1 µM CellTracker CMTMR (Invitrogen. Carlsbad, California) for 15 min, washed with RPMI-10%FBS, incubated for 2 h in RPMI-10%FBS, and washed again in RPMI-10%FBS. Infected donor and CMTMR-stained target cells were cocultured in 0.5 ml RPMI-10%FBS for 3 h in the presence or absence of the myosin light chain kinase (MLCK) inhibitor, ML7 (40 µM) (EMD Biosciences. San Diego, California), or the solvent negative control DMSO. The CD4 blocking antibody Leu3A (0.25 µg/ml) (BD Biosciences) and isotype anti-mouse IgG control antibody (0.25 µg/ml) (Santa Cruz Biotechnology. Santa Cruz, California) were utilized to validate the assay, as it was shown previously using a similar assay that virus transfer was dependent on Env-CD4 interaction [53](Figure 4A). To rule out the possibility that inhibitors affect viral protein synthesis and thereby indirectly alter virus transfer, 10 µg/ml cycloheximide, which abolishes protein synthesis, was added at the beginning of coculture. After 3 h of coculture, cells were fixed in 4% PFA. Double-positive cells, which represent CMTMR-positive target cells that received YFP-containing virus particles, were identified by flow cytometry (see Figure 4A for examples). Results were presented as a percentage of double-positive cells compared to total CMTMR-stained target cells.

### Quantification of polarization

To measure morphological polarization of T cells, outlines of Gag-YFP-expressing P2 cells were determined by manually tracing the cell perimeter using the ImageJ program. Circularity values were then calculated based on this outline using the Measure function of ImageJ. The output values range between 0 and 1, with 1 representing a perfect circle. This method has been described previously [168]. Morphologically polarized cells with circularity values below 0.8 were further examined for polarization of Gag localization. To quantify polarity of Gag localization, a 10-segmented grid was placed over each cell along the cell's longest axis. The number of segments that contained plasma-membrane-associated Gag was then used as the polarization index. Lower values correspond to more polarity of Gag on the cell surface. Examples of these quantifications are shown in Figure S4.

## Supporting Information

Text S1 This file includes Supplementary Materials and Methods and Supplementary Discussion.
(0.06 MB DOC)

Figure S1 Untagged Gag detected at the plasma membrane using anti-Gag antibodies shows strong colocalization with uropod markers. P2 cells infected with wild type HIV-1 (NL4-3) were labeled with anti-PSGL-1 or anti-CD43 (red) prior to fixation. Fixed cells were permeabilized and immunostained with anti-p17MA or anti-p24CA (green). Note that when Gag is detected on the cell surface, it colocalized with uropod markers.
(4.17 MB TIF)

Figure S2 Effects of ML7 on VLP release efficiency. P2 cells expressing Gag-YFP or Fyn(10)delNC Gag-YFP were metabolically labeled with [^35^S] methionine/cysteine for 2 h in the presence of 40 µM ML7 (+) or DMSO (-). Cell and virus lysates were subjected to immunoprecipitation of viral proteins using HIV-Ig. Virus release efficiency was calculated as the amount of virion-associated Gag as a fraction of total (cell plus virion) Gag synthesized during a 2-h metabolic labeling period.
(6.93 MB TIF)

Figure S3 Depolarization of T cells by ML7 treatment reduces cell-to-cell transfer of virus particles. A) Transfer of Gag-YFP fluorescence from infected P2 cells to CMTMR-stained SupT1 target cells during a 3-h coculture period was measured by flow cytometry. ML7, DMSO, or antibodies, along with 10 µg/ml cycloheximide, were added at the beginning of the coculture period. Flow cytometry plots are shown. Gate A, CMTMR-labeled target cells; gate B, double positive cells representing target cells with transferred Gag-YFP particles; and gate C, YFP-expressing cells either fused or conjugated to CMTMR-labeled target cells. B) Representative brightfield (top panels) and fluorescence (bottom panels) images of cocultures untreated or treated with DMSO or ML7 are shown. Gag-YFP and CMTMR signals were shown in green and red, respectively.
(9.96 MB TIF)

Figure S4 Examples of polarity index calculations. To measure morphological polarization of T cells, outlines of Gag-YFP-expressing P2 cells were determined by manually tracing the cell perimeter using the ImageJ program. Circularity values were then calculated based on this outline using the Measure function of ImageJ. The output values range between 0 and 1, with 1 representing a perfect circle. To quantify polarity of Gag localization, a 10-segmented grid was placed over each cell along the cell's longest axis. The number of segments that contained plasma-membrane-associated Gag was then used as the polarization index. For clarity, the outline and the grid were removed from the lower right panel.
(7.90 MB TIF)

Movie S1 Migrating T cell stably maintains uropod localization of Gag. Cells expressing Gag-YFP (green) were immunostained with anti-PSGL-1 prelabeled by AlexaFluor-594-conjugated anti-mouse IgG (red). Images were acquired every 30 s for 30 min as the polarized cell migrates through the field. Yellow color indicates colocalization of PSGL-1 and Gag-YFP.
(3.13 MB MOV)

Movie S2 Infected T cells mediate stable contacts with target cells via their uropods. Primary T cells expressing Gag-YFP (green) were cocultured with fresh primary T cells from the same donor stained with the fluorescent dye CMAC (blue) and immunostained with an anti-PSGL-1 antibody (red) as described in Materials and Methods. Regions of colocalization between Gag and PSGL-1 are shown in yellow. Live cell images were taken every 30 s for 20 min. Note that the uropod, enriched in Gag-YFP and PSGL-1, mediates stable contacts with target cells.
(2.75 MB MOV)

Movie S3 Cell surface patches containing Gag and a uropod marker laterally move to and accumulate at a forming uropod. Time lapse images of a Gag-YFP-expressing T cell during repolarization. Gag-YFP(green)-expressing P2 cells were immunostained for PSGL-1 (red) as described in Figure 6. Cells were then depolarized by incubation at 4°C for 30 min. Approximately 5 min after chamber coverslips containing depolarized cells were transferred to the microscope stage maintained at 37°C, acquisition of live cell images at 30-s intervals was begun and continued for 27 min. Note that the small patches migrate and coalesce to the large patch at the cell pole that eventually forms the uropod.
(1.55 MB MOV)

Movie S4 Gag puncta move to and accumulate at a forming uropod in the absence of a crosslinking antibody. Time lapse images of a Gag-YFP-expressing T cell during repolarization. Gag-YFP(green)-expressing P2 cells were depolarized by incubation at 4°C for 30 min. Approximately 5 min after chamber coverslips containing depolarized cells were transferred to the microscope stage maintained at 37°C, acquisition of live cell images at 30-s intervals was begun and continued for 26 min. Note that even as the cell rotates and changes direction, the small patches migrate and coalesce to the large patch at the cell pole that eventually forms the uropod.
(4.69 MB AVI)
