# Supplementary material for: Nucleocapsid Promotes Localization of HIV-1 Gag to Uropods That Participate in Virological Synapses between T Cells
Source: PLoS Pathog. 2010 Oct 28;6(10):e1001167. doi: 10.1371/journal.ppat.1001167 (PMC2965768; doi:10.1371/journal.ppat.1001167)
Supplement: Text S1 — This file includes Supplementary Materials and Methods and Supplementary Discussion. (0.06 MB DOC) [file ppat.1001167.s001.doc]

# Supplementary Text

**Supplementary Materials and Methods**

**Immunostaining.** To assess localization of untagged Gag, P2 cells infected with wild type HIV-1 (NL4-3) were incubated with anti-PSGL-1 or anti-CD43 prelabeled with Zenon AlexaFluor 594 (Invitrogen) for 30 min at 37ºC, washed with RPMI-10%FBS, and further cultured for 30 min prior to fixation with 4% PFA. Fixed cells were permeabilized by 10-min incubation in PBS containing 0.2% saponin and 5% FBS (PBS-saponin-FBS) prior to 30-min incubation with Zenon AlexaFluor488-labeled mouse monoclonal anti-p17MA (Applied Biotechnologies) or anti-p24 (clone 183-H12-5C; AIDS Research and Reference Reagent Program). Cells were subsequently washed with PBS-saponin-FBS, fixed with 4% PFA for 10 min, washed with PBS-2%FBS, and mounted as described in the main text. The monoclonal anti-p24 used in this study preferentially recognizes cytosolic Gag as observed for other anti-CA antibodies [1]. As a result, with this antibody, we observed that only approximately 5% of Gag-positive cells showed Gag signal on the cell surface, and the rest displayed only hazy cytosolic signal. Nonetheless, when Gag is detected on the cell surface, it colocalized with uropod markers.

**Virus Release Assay.** 3x10^5^ P2 cells were infected with VSV-G-pseudotyped HIV-1 expressing Gag-YFP. Two days post-infection, metabolic labeling of infected cells with [^35^S] Met/Cys, preparation of cell lysates, and immunoprecipitation of viral proteins using HIV immunoglobulin (HIV-Ig; AIDS Research and Reference Reagent Program) were performed as described previously [2]. Virus release efficiency was calculated as the amount of virion-associated Gag as a fraction of total (cell plus virion) Gag synthesized during a 2-h metabolic labeling period.

**Supplementary Discussion on Cell-to-Cell Virus Transfer Experiments**

The finding that ML7, an inhibitor of cell polarization, reduces virus transfer is consistent with a role for uropods in formation of the VS and cell-to-cell transfer. However, although ML7 does not affect virus particle production efficiency (Figure S2 panel A), we cannot exclude potential pleiotropic effects of this inhibitor on cellular functions such as cell motility or cell adhesion. As for cell motility, cell-to-cell transfer was examined using highly dense cocultures (2.6x10^6^/ml). Therefore, even though ML7-treated cells have little motility, passive cell-cell contacts occur (Figure S2 panel B). As for cell adhesion, however, this high density of cocultures prevented us from identifying cell conjugates and quantitatively assessing effects of ML7 on the cell conjugate stability. Therefore, we cannot rule out the possibility that ML7 inhibits virus transfer not directly through depolarization of viral protein localization but through other effects such as inhibition of stable cell adhesion, which may nonetheless diminish the VS stability. Yet, it is of note that, even if ML7 inhibits stable conjugation between infected and target cells, such effect may still be caused by disruption of uropods, as uropods are enriched in adhesion molecules [3]. Moreover, our data indicating that Gag- and Env-containing uropods or subcomponents of such uropods constitute the VS (Figures 2-4) strongly suggest the role for uropods in cell-to-cell virus transfer, as the VS likely facilitates cell-to-cell virus transfer [4].

In an attempt to address genetically the importance of uropods in cell-to-cell virus transfer, we sought to analyze the VLP transfer of cells expressing NC mutant Gag proteins. As NC mutants localize over the entire cell surface and do not specifically accumulate to uropods unlike WT Gag, one might predict that these mutants are inefficient in cell-to-cell transfer if Gag accumulation to the uropod, and eventually to the VS, plays a key role in cell-to-cell transfer. We did observe reduction in transfer of Fyn(10)delNC Gag from donor to target cells (data not shown). However, we also observed that NC deletion has pleiotropic impacts on Gag particle production, which complicate the interpretation of the transfer experiment. First, VLP release efficiency by Fyn(10)delNC Gag is 2 fold less than that by WT Gag (Figure S2). Second, ML7 does not affect virus release efficiency of WT Gag, whereas the efficiency of Gag particle release by the Fyn(10)delNC mutant is drastically increased by ML7 (Figure S2), suggesting that different cellular factors are involved in particle production of the NC mutant Gag. Third, in electron microscopy (EM) of transfected HeLa cells, we observed that unlike WT Gag, Fyn(10)delNC Gag gave rise to thin protrusions and folding of the plasma membrane lined by an electron dense layer, presumably formed by this NC-deleted Gag derivative (data not shown). Similar EM phenotypes were previously reported for Gag truncation mutants lacking NC [5, 6]. Altogether, these differences indicate that WT and Fyn(10)delNC Gag form and release extracellular particles in different manners. Because of this complicating factor, we concluded that results of experiments that compare WT and mutant Gag proteins for cell-to-cell transfer are not interpretable.

## References

1. Hubner W, Chen P, Del Portillo A, Liu Y, Gordon RE, et al. (2007) Sequence of human immunodeficiency virus type 1 (HIV-1) Gag localization and oligomerization monitored with live confocal imaging of a replication-competent, fluorescently tagged HIV-1. J Virol 81: 12596-12607.
2. Ono A, Ablan SD, Lockett SJ, Nagashima K, Freed EO (2004) Phosphatidylinositol (4,5) bisphosphate regulates HIV-1 Gag targeting to the plasma membrane. Proc Natl Acad Sci U S A 101: 14889-14894.
3. Sanchez-Madrid F, Serrador JM (2009) Bringing up the rear: defining the roles of the uropod. Nat Rev Mol Cell Biol 10: 353-359.
4. Jolly C, Kashefi K, Hollinshead M, Sattentau QJ (2004) HIV-1 cell to cell transfer across an Env-induced, actin-dependent synapse. J Exp Med 199: 283-293.
5. Gheysen D, Jacobs E, de Foresta F, Thiriart C, Francotte M, Thines D, De Wilde M (1989) Assembly and release of HIV-1 precursor Pr55gag virus-like particles from recombinant baculovirus-infected insect cells. Cell. 59:103-112.
6. Jowett JB, Hockley DJ, Nermut MV, Jones IM (1992) Distinct signals in human immunodeficiency virus type 1 Pr55 necessary for RNA binding and particle formation. J Gen Virol. 73:3079-3086.
