# Supplementary figures and images for: Nucleocapsid Promotes Localization of HIV-1 Gag to Uropods That Participate in Virological Synapses between T Cells
Source: PLoS Pathog. 2010 Oct 28;6(10):e1001167. doi: 10.1371/journal.ppat.1001167 (PMC2965768; doi:10.1371/journal.ppat.1001167)

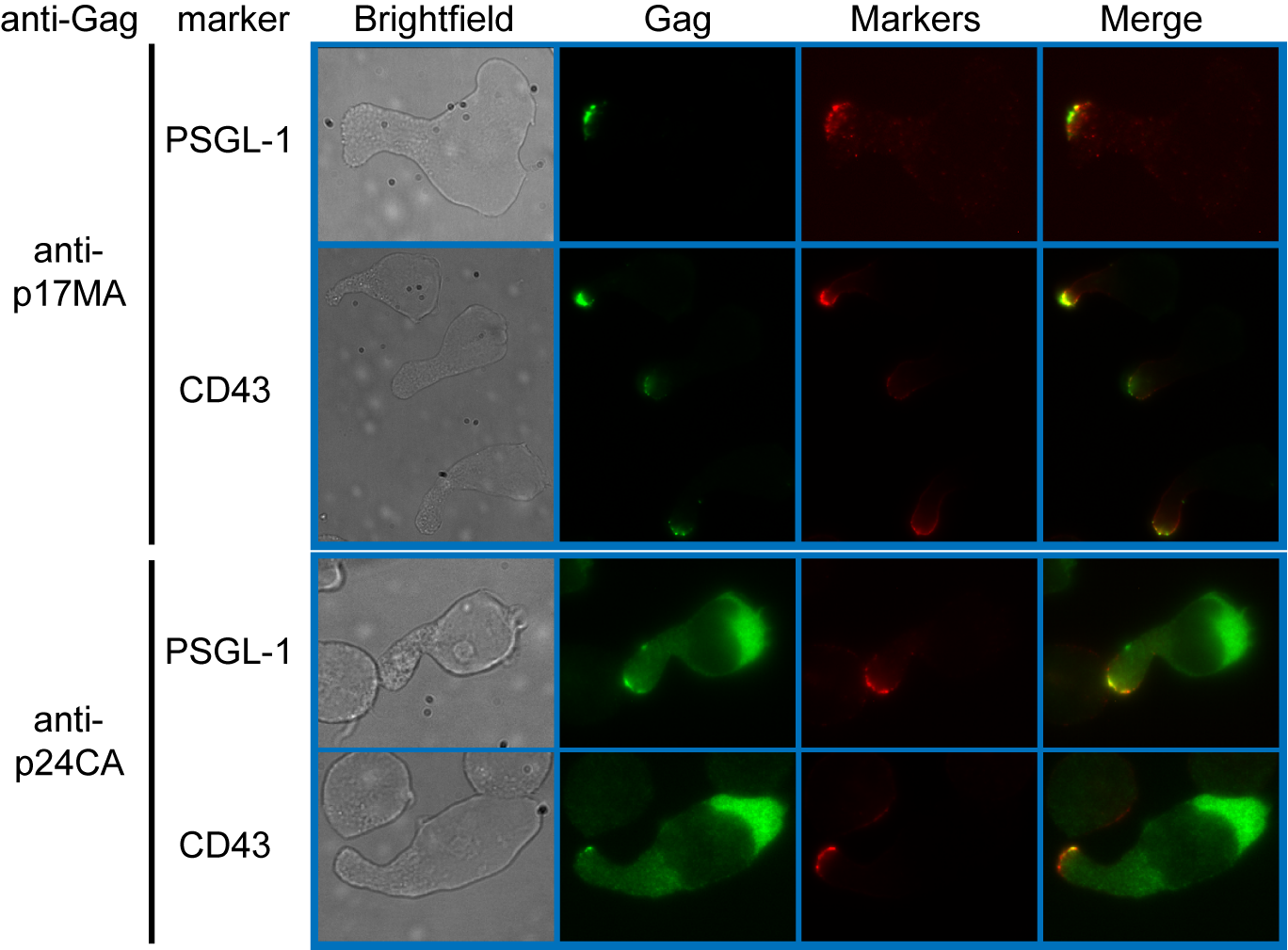

Supplement: Figure S1 — Untagged Gag detected at the plasma membrane using anti-Gag antibodies shows strong colocalization with uropod markers. P2 cells infected with wild type HIV-1 (NL4-3) were labeled with anti-PSGL-1 or anti-CD43 (red) prior to fixation. Fixed cells were permeabilized and immunostained with anti-p17MA or anti-p24CA (green). Note that when Gag is detected on the cell surface, it colocalized with uropod markers. (4.17 MB TIF) [file ppat.1001167.s002.tif]

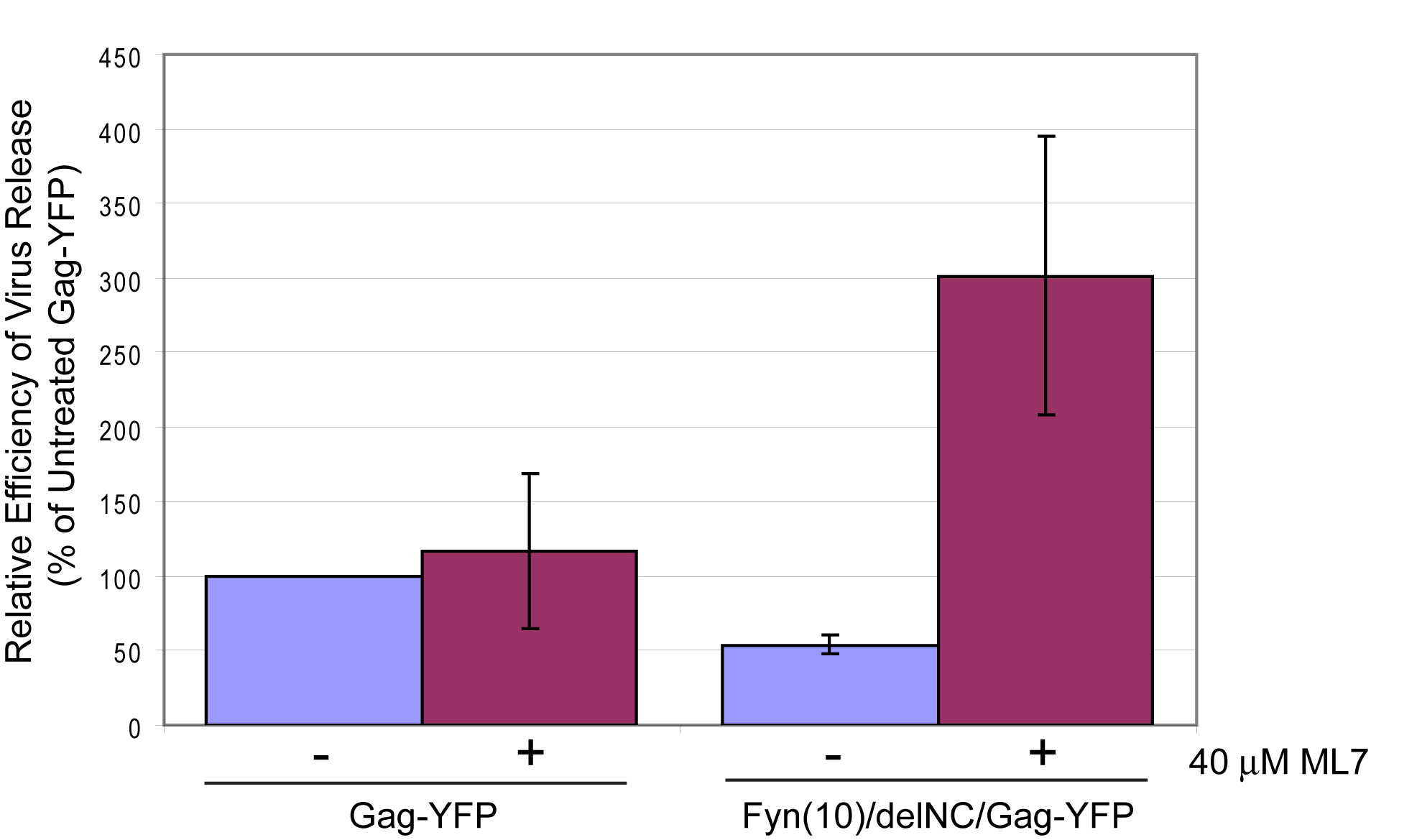

Supplement: Figure S2 — Effects of ML7 on VLP release efficiency. P2 cells expressing Gag-YFP or Fyn(10)delNC Gag-YFP were metabolically labeled with [35S] methionine/cysteine for 2 h in the presence of 40 µM ML7 (+) or DMSO (-). Cell and virus lysates were subjected to immunoprecipitation of viral proteins using HIV-Ig. Virus release efficiency was calculated as the amount of virion-associated Gag as a fraction of total (cell plus virion) Gag synthesized during a 2-h metabolic labeling period. (6.93 MB TIF) [file ppat.1001167.s003.tif]

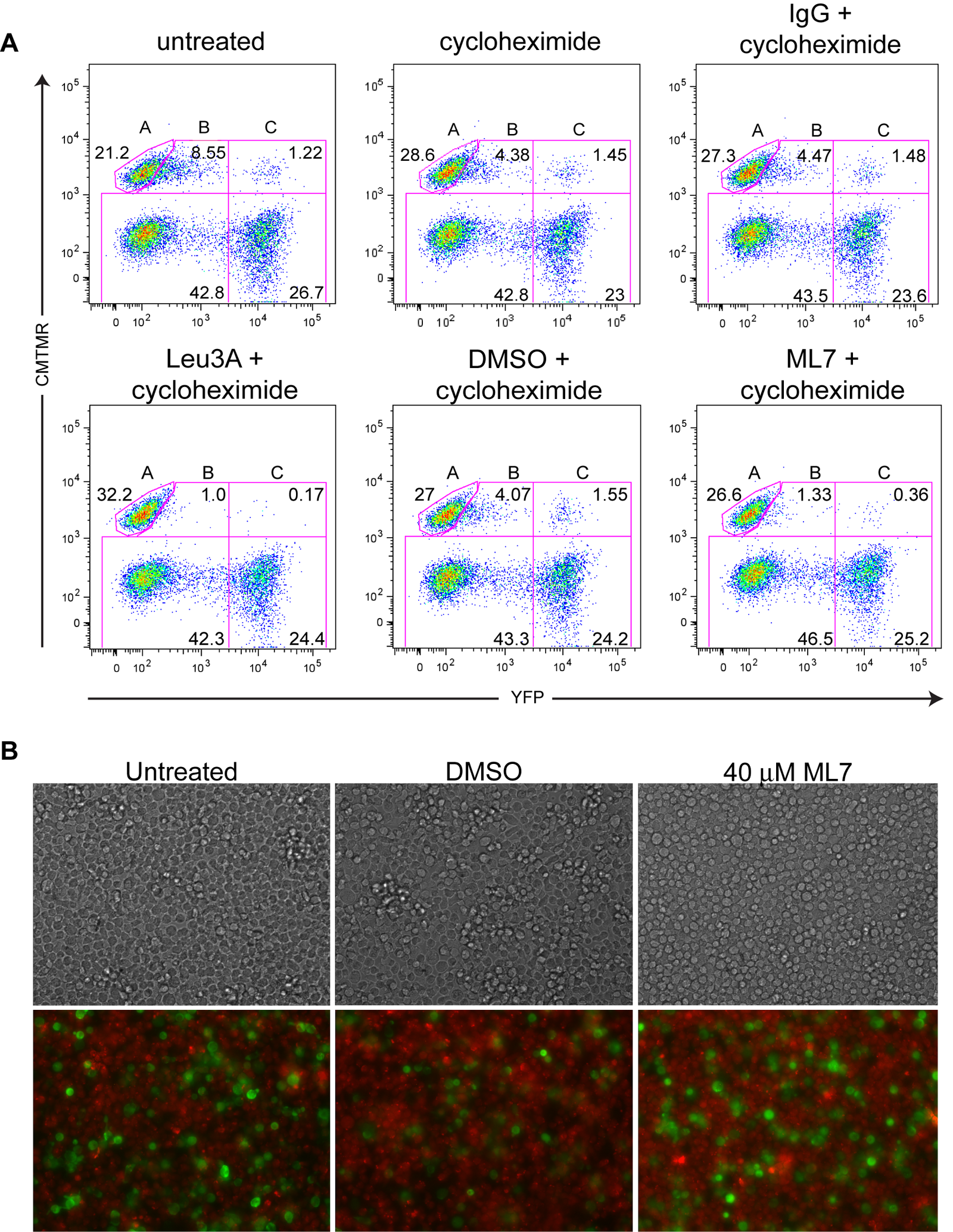

Supplement: Figure S3 — Depolarization of T cells by ML7 treatment reduces cell-to-cell transfer of virus particles. A) Transfer of Gag-YFP fluorescence from infected P2 cells to CMTMR-stained SupT1 target cells during a 3-h coculture period was measured by flow cytometry. ML7, DMSO, or antibodies, along with 10 µg/ml cycloheximide, were added at the beginning of the coculture period. Flow cytometry plots are shown. Gate A, CMTMR-labeled target cells; gate B, double positive cells representing target cells with transferred Gag-YFP particles; and gate C, YFP-expressing cells either fused or conjugated to CMTMR-labeled target cells. B) Representative brightfield (top panels) and fluorescence (bottom panels) images of cocultures untreated or treated with DMSO or ML7 are shown. Gag-YFP and CMTMR signals were shown in green and red, respectively. (9.96 MB TIF) [file ppat.1001167.s004.tif]

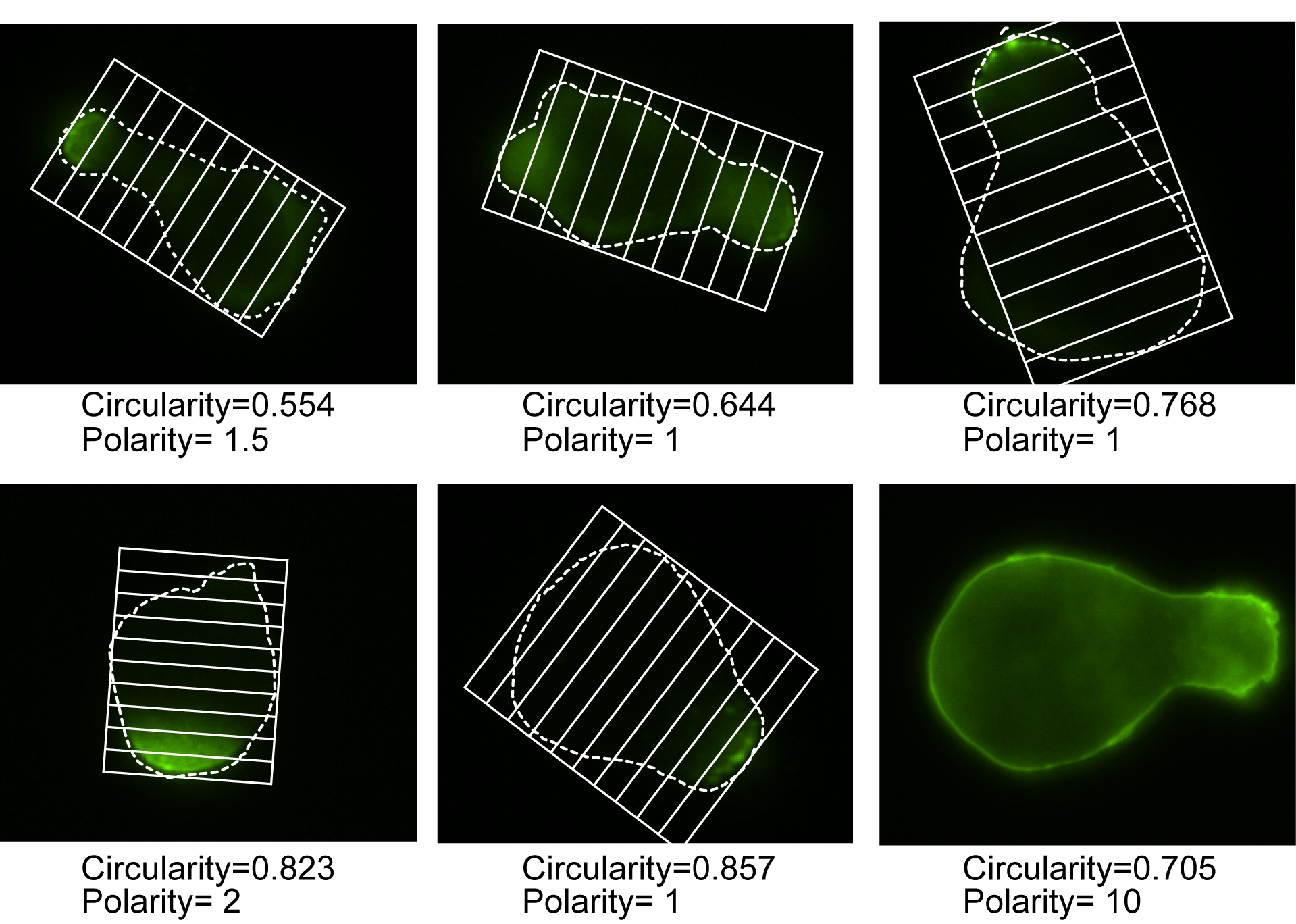

Supplement: Figure S4 — Examples of polarity index calculations. To measure morphological polarization of T cells, outlines of Gag-YFP-expressing P2 cells were determined by manually tracing the cell perimeter using the ImageJ program. Circularity values were then calculated based on this outline using the Measure function of ImageJ. The output values range between 0 and 1, with 1 representing a perfect circle. To quantify polarity of Gag localization, a 10-segmented grid was placed over each cell along the cell's longest axis. The number of segments that contained plasma-membrane-associated Gag was then used as the polarization index. For clarity, the outline and the grid were removed from the lower right panel. (7.90 MB TIF) [file ppat.1001167.s005.tif]
